# Artificial lagoon project alters archaeal diversity, community assembly, and potential activity around a nearshore island: insights from an annual cycle

**DOI:** 10.1128/aem.01499-25

**Published:** 2025-12-30

**Authors:** Haoyu Song, Xuya Hu, Zhen Chen, Lanying Yuan, Pengbo Gao, Yujie Huang, Demin Zhang, Kai Wang

**Affiliations:** 1 Key Laboratory of Aquacultural Biotechnology, Ministry of Education, at School of Marine Sciences, Ningbo University 47862, Ningbo, China; 2 Meishan Bay Tourism Development Service Center in Beilun District, Ningbo, China; 3 Collaborative Innovation Center for Zhejiang Marine High-efficiency and Healthy Aquaculture, Ningbo University 47862, Ningbo, China; University of Illinois Urbana-Champaign, Urbana, Illinois, USA

**Keywords:** coastal lagoon, planktonic archaea, community assembly mechanism, microbial activity, water-quality restoration

## Abstract

Coastal lagoon project is a common strategy for enhancing flood control capability and ecosystem services, yet its impact on microbiota, especially archaea, remains unclear. Using 16S rRNA gene and transcript sequencing, we compared archaeal diversity, community assembly processes, and potential activity in an artificial lagoon and adjacent seaward waters through monthly sampling over an annual cycle. The lagoon has created a distinct water environment with reduced salinity and turbidity, along with unique dissolved organic matter profiles. The lagoon’s influence overrode seasonal variability in archaeal alpha-diversity, yielding overall higher levels within the lagoon. Despite pronounced seasonal shifts—Nitrosopumilaceae dominating in cooler seasons and Poseidoniales prevailing in warmer months—the lagoon’s influence on archaeal community composition across taxonomic scales remained evident, particularly in the spatial niche partitioning of Poseidoniales populations. Lagoon archaeal communities exhibited higher turnover rates and accelerated seasonal recurrence compared with those in the seaward zone. Although archaeal community assembly was primarily driven by water-mass effects, selection occasionally exerted a stronger influence in seaward waters. Analyses involving the 50 most abundant zero-radius operational taxonomic units (ZOTUs) revealed that the lagoon project had a stronger and more widespread effect on the distribution of key archaeal taxa than on their potential activity, consistent with the trend observed at the genus level, except for two Nitrosopumilaceae genera: *Nitrosopumilus* often exhibited lower activity, while *Nitrosopelagicus* occasionally showed higher activity in the lagoon. Our findings highlight that the lagoon project variably altered archaeal diversity, community assembly, and potential activity, underscoring microbial consequences and potential ecological impacts of nearshore restoration projects.

**IMPORTANCE:**

Coastal lagoon projects are widely employed to enhance ecosystem services, such as water quality, yet their impacts on microbial communities—particularly archaea—remain poorly understood. This year-long study reveals that artificial lagoon environments significantly reshape archaeal communities by increasing alpha-diversity, accelerating seasonal turnover, and shifting dominant taxa, especially among ammonia-oxidizing archaea and Poseidoniales. Community assembly was primarily governed by water-mass effects introduced through lagoon maintenance, while archaeal potential activity exhibited taxon-specific patterns. These findings uncover critical, previously overlooked microbial consequences of lagoon engineering and emphasize the importance of incorporating microbial dynamics into the planning and evaluation of nearshore restoration projects.

## INTRODUCTION

Although planktonic archaea were first discovered in coastal marine waters (1, 2), our understanding of the diversity, assembly, and activity of archaeal communities in these environments remains limited, even after decades of research (3). This is largely due to the highly diverse and variable environmental conditions of coastal ecosystems. One major challenge in uncovering patterns in archaeal communities in coastal environments is the limited knowledge of how anthropogenic impacts influence their dynamics. Human activities introduce complex and intensive disturbances, further complicating efforts to study these communities.

Coastal lagoons are shallow channels or ponds that communicate with open marine water bodies, performing critical ecosystem service functions, such as water quality maintenance, flood control, fisheries, nutrient retention and export, and carbon fixation and sequestration, in the transitional zone between land and sea (4). Despite their ecological importance, these systems face numerous threats, including overexploitation, habitat destruction, terrestrial emissions, agricultural and industrial runoff, and tourism activities, all of which contribute to their increasing vulnerability (5). To enhance ecosystem services or to mitigate ecological vulnerabilities, coastal lagoons are often subject to artificial modifications, such as dam construction or the intentional opening of sand barriers to regulate their connection with open marine waters (6, 7). In these anthropogenically impacted ecosystems, it is crucial to assess how modified environmental conditions—such as changes in salinity, nutrient levels, and dissolved organic matter (DOM) compared to adjacent open waters—affect the diversity, assembly, and activity of microorganisms. Such assessments are vital to understanding the ecological impacts of anthropogenic disturbances (8). While many efforts have been made to investigate the compositional variation of bacterial and microeukaryotic communities in the water and sediments of coastal lagoons (5, 9–11), relatively few studies have focused on the unique microbial profiles of lagoons compared to adjacent open waters (12, 13). Even less is known about the dynamics and divergences of archaeal communities in coastal lagoons and their adjacent waters.

Seasonal dynamics of major planktonic archaeal groups, such as Thaumarchaeota Marine Group (MG) I (within the family Nitrosopumilaceae according to GTDB taxonomy [14])—which serves as the primary ammonia oxidizer (ammonia-oxidizing archaea, AOA) and ranks among the most abundant organisms in marine environments—have been observed at the SPOT station in the coastal waters of Southern California (15, 16). Another key group is Euryarchaeota MGII (now classified within the order Poseidoniales), a globally abundant group of (photo)heterotrophic organisms supported by genomic evidence (17–19). These studies have further explored their diversified ecotypes (16, 18, 19), revealing their niche partitioning patterns across the water column, as well as the abiotic and biotic factors shaping these patterns (15, 16). Compared to studies on diversity and composition, much less attention has been paid to the activity of planktonic archaea. A notable study utilizing 16S rRNA to 16S rRNA gene (RNA:DNA) sequence ratios demonstrated seasonal variations in the activity of different ecotypes of MGI, MGIIa (now Poseidoniaceae), and MGIIb (now Thalassarchaeaceae), at the SOLA station in Banyuls-sur-Mer Bay in the Mediterranean Sea (20). However, most prior research on archaeal dynamics in coastal waters has been conducted at single stations, often focusing on oligotrophic waters with minimal human impact. As a result, comparisons between stations experiencing distinct anthropogenic influences remain scarce.

**Fig 1:**
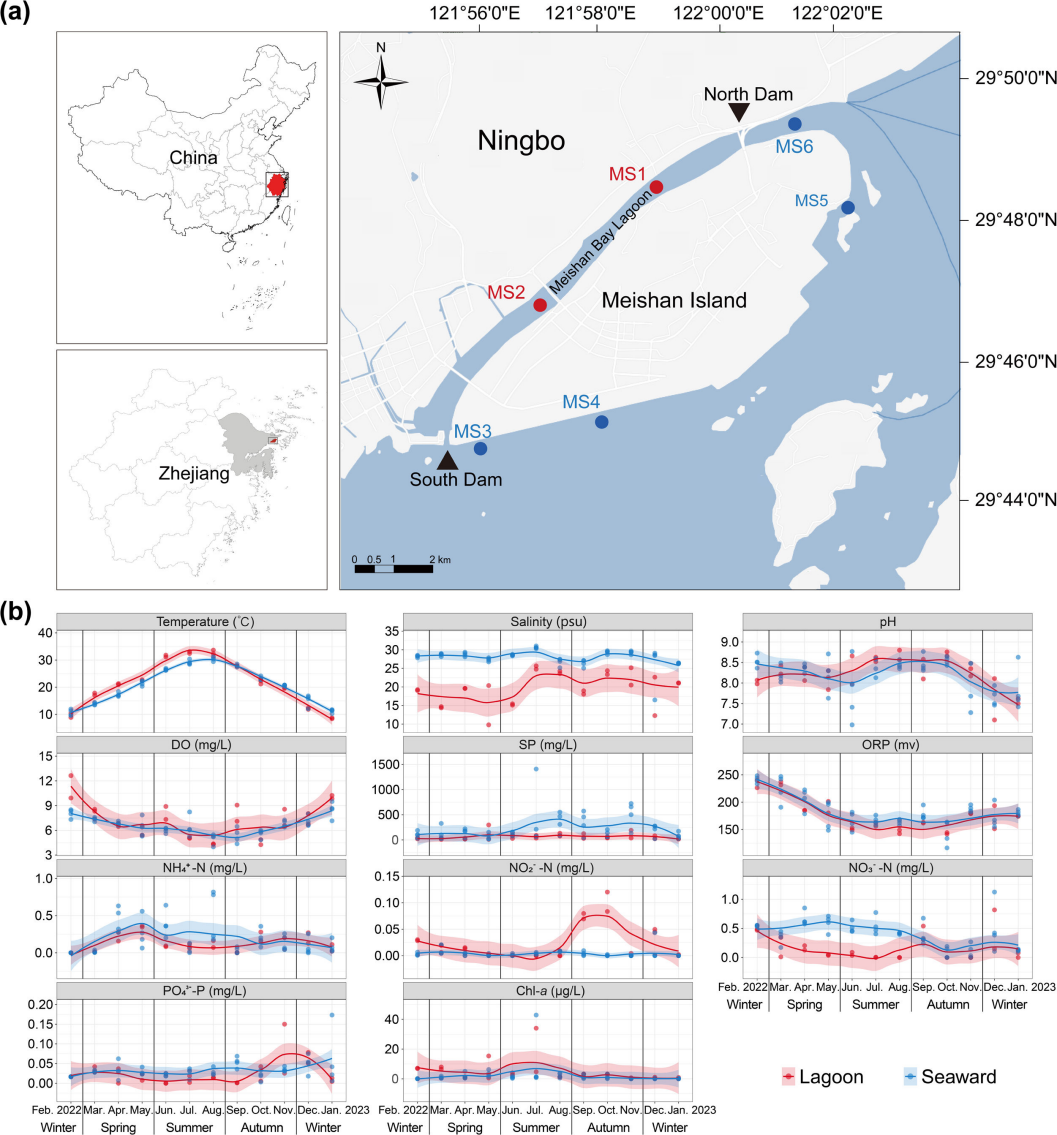
The map of sampling stations around the Meishan Island (**a**). Stations in red and blue represent Meishan Bay Lagoon (Lagoon) and adjacent waters (Seaward), respectively. The map was generated using ArcGIS. Annual dynamics of environmental factors in the lagoon and seaward waters (**b**). DO, dissolved oxygen; SP, suspended particles; ORP, oxidation-reduction potential; Chl-*a*, chlorophyll *a*. For a given parameter, a value of zero was recorded when its concentration fell below the detection limit in a specific sample. The shaded areas represent the 95% confidence intervals for the fitted LOESS curves.

Our study focuses on an artificially enhanced lagoon and its adjacent waters around Meishan Island in the East China Sea, located approximately 500 m from the mainland (Fig. 1a). The lagoon, officially named Meishan Bay (hereafter referred to as Meishan Bay Lagoon), spans about 10 km^2^ and consists of a narrow, landward water channel with dams constructed at both ends. The North Dam was completed in October 2013 and the South Dam in May 2016, serving as artificial inlets and outlets. These modifications have transformed the lagoon into a semi-enclosed system, creating distinct environmental conditions compared to the adjacent waters (hereafter referred to as seaward zone) (21). Meishan Bay Lagoon is primarily maintained for flood control, water quality improvement, and ecological restoration, while also receiving riverine discharge from the neighboring area, placing it under certain anthropogenic influence. In contrast, the seaward side of the island functions as a hub for Ningbo-Zhoushan Port, one of the world’s largest ports, exposing the adjacent waters to frequent shipping activities. The contrasting types of human activity across the two zones provide an ideal model system for assessing the impact of artificial lagoon projects on planktonic archaeal diversity, community assembly, and activity over the seasonal cycle.

Our previous research has identified archaea as a key component of the microbiome in the waters around Meishan Island, accounting for 0.6%–26.5% of the prokaryotic community (21). In this study, we set representative stations in both the lagoon and the seaward zone and conducted a year-long monthly observation. To characterize total and potentially active archaeal assemblages, we sequenced the 16S rRNA gene and its transcript (16S rRNA). Our major aims were (i) to compare the total and potentially active archaeal assemblages between the lagoon and the seaward zone across different seasons; (ii) to assess shifts in the relative importance of water-mass effect and species selection in shaping archaeal community assembly across the two zones and seasons by analyzing the convergence and divergence between DNA- and RNA-based assemblages; (iii) to infer the potential activity of key archaeal taxa between the two zones over the seasonal cycle. This work provides a comprehensive assessment of how a coastal artificial lagoon project influences diversity, community assembly, and potential activity of planktonic archaea from a seasonal perspective.

## MATERIALS AND METHODS

### Study area, lagoon maintenance, sampling scheme, and water physicochemical analyses

The main municipal functions of Meishan Bay Lagoon are flood control and ecological restoration. Its routine maintenance follows the basic principle of maintaining the water level–salinity balance to ensure ecosystem stability. To achieve this, water is typically inlet through the North Dam (bringing in higher-salinity seawater from the open sea) and discharged through the South Dam (releasing lower-salinity water from inside the lagoon), which together regulate both water level and salinity. These operations are adjusted according to weather conditions (primarily rainfall) and water levels and can be divided into three main stages: (i) Medium-frequency regulation stage (February–May): a total of 43 regulation events were conducted, averaging five water inlets per month (total volume: 38.84 million m³) and about five discharges per month (total volume: 59.86 million m³), with a water level fluctuation of 0.01–0.66 m (in elevation). During this stage, rainfall was occasional and relatively stable, resulting in minor water level variations. The primary regulation goal was to maintain the basic water level while reserving storage capacity for the upcoming rainy season. (ii) High-frequency intervention stage (June–September): a total of 121 regulation events were conducted, averaging about 12 water inlets per month (total volume: 76.84 million m³) and about 17 discharges per month (total volume: 101.04 million m³), with a water level fluctuation of −0.50–0.94 m. This stage was the most frequent and intensive regulation period of the year and mainly influenced by increased rainfall during the rainy season (June and July) and later typhoon surges (August and September), which significantly amplified lagoon water level fluctuations. The operational goals focused on maintaining water level safety, enhancing water exchange, and improving water quality regulation. (iii) Low-frequency maintenance stage (October–December): a total of 19 regulation events were conducted, averaging about three water inlets per month (total volume: 15.32 million m³) and two discharges per month (total volume: 14.98 million m³), with a water level fluctuation of 0.00–1.01 m. This stage corresponds to the dry season, characterized by reduced rainfall and low hydrological disturbance in the lagoon.

Six stations were set around the Meishan Island, with two stations in the Meishan Bay Lagoon (Lagoon stations) and four stations in the adjacent waters (Seaward stations) (Fig. 1a). Monthly observations were conducted over a 1-year period, from February 2022 to January 2023. Surface water (~0–0.5 m) of each station was sampled. Subsamples for DNA and RNA extraction were preserved in RNAlater, filtered onto a 0.2-μm polycarbonate membrane (Millipore, USA), and then stored at −80°C. Water temperature, pH, dissolved oxygen (DO), and salinity were measured *in situ* using a YSI 550A probe (YSI, USA), and oxidation-reduction potential (ORP) was measured using a CT-8022 mini ORP meter (Keddie, China). Ammonium (NH_4_^+^), nitrate (NO_3_^−^), nitrite (NO_2_^−^), phosphate (PO_4_^3−^), chlorophyll *a* (Chl-*a*), and suspended particles (SP) were measured with the standard methods (22). A detailed description of the methodologies for quantifying fluorescent dissolved organic matter (FDOM) is provided in the Supplemental methods.

### Nucleic acid extraction, PCR amplification, and 16S rRNA amplicon sequencing

Total DNA and RNA on the filters were extracted using an All Prep DNA/RNA Micro Kit (Qiagen, Germany) following the manufacturer’s instructions. Purified RNA samples were reverse transcribed into cDNA using the SuperScript IV First-Strand Synthesis System Kit (Thermo Fisher Scientific, USA). The V4–V5 region of archaeal 16S rRNA genes was amplified for DNA and cDNA using the dual-indexed primer set: 524F (5′-TGYCAGCCGCCGCGGTAA-3′) and 958R (5′-YCCGGCGTTGAVTCCAATT-3′) (23). An amount of approximately 50 ng purified DNA or cDNA template from each sample was amplified in a 50-μL reaction system under the following conditions: initial denaturation at 94°C for 5 min; then 35 cycles of denaturation at 94°C for 30 s, annealing at 53°C for 30 s, and extension at 72°C for 30 s; with a final extension at 72°C for 8 min. The pooled PCR products were purified using the E.Z.N.A. Gel Extraction Kit (Omega, USA). The library was prepared using the ALFA-SEQ DNA Library Prep Kit (Findrop, China) and quantified with a Qubit 4.0 fluorometer (Thermo Fisher Scientific, USA). Sequencing was performed using a NovaSeq 6000 platform (Illumina, USA). A detailed description of the methodologies for sequence processing is provided in the Supplemental methods.

### Measurement of active archaeal community and potential activity of archaea

We used 16S rRNA gene and 16S rRNA (cDNA) sequences to present the total archaeal community and potentially active assemblages, respectively (24, 25). The 16S rRNA to 16S rRNA gene (RNA:DNA) sequence ratios (hereafter referred to as 16S ratios) were used to estimate *in situ* potential activity trends (26) across taxonomic scales, encompassing genus-level taxa, as well as the top 50 most abundant zero-radius operational taxonomic units (ZOTUs), accounting for 84.62% of archaeal sequences in the entire data set. To address the challenge of “phantom taxa”—taxa detected in RNA but not in DNA, which result in a zero denominator for the 16S ratio—we replaced all observations with RNA > 0 and DNA = 0 with DNA = 1 in the ZOTU table prior to normalization by proportions and subsequent calculation of 16S ratios, as previously suggested (27).

### Inference of dominant assembly processes of archaeal community

A framework based on the convergence and divergence patterns between DNA- and RNA-based microbial assemblages was applied to infer shifts in the relative importance of species selection and water-mass effect (indicating dispersal processes) in shaping archaeal community assembly between the lagoon and adjacent waters as previously described (27). Briefly, we firstly calculated the distance among DNA-RNA sample pairs (within the n-dimensional space computed from the principal coordinate analysis [PCoA] ordination space) across n_75%_ axes—first axes that cumulatively explain 75% of the variation for each ordination (28), resulting in both Bray-Curtis distance (m_BC_) and Sørensen distance (m_S_), and then the difference between m_BC_ and m_S_ (Δ-distance) was calculated to examine the relative importance between abundance-based and incidence-based distances. Lower or negative Δ-distance values indicate relatively higher incidence-based distances, suggesting dissimilarity is largely driven by the presence of different taxa between DNA- and RNA-based assemblages and can thus be regarded as a process dominated by water-mass effects. On the contrary, higher Δ-distance values indicate relatively low incidence-based distance and high abundance-based distance, suggesting selection-driven dissimilarity with more taxa in common but large numerical differences between DNA-based and RNA-based assemblages, and can thus be regarded as a selection-dominated process (27). As tested and indicated by the framework developers (27), absolute values of Δ-distances hold limited meaning, and the relative change in Δ-distances and the resulting pattern between the two zones or over time are informative and comparable.

### General statistical analyses

Principal component analysis (PCA) was applied to visualize the compositional variation of FDOM across months or zones using the R package “stats” (29). The alpha-diversity indices (including ZOTU richness, Shannon index, and phylogenetic diversity) were calculated using the R packages “vegan” and “picante” (30, 31). Three-way repeated measures analysis of variance (ANOVA) was applied to test the influence of month, zone, nucleic acid type, and their interactions on archaeal alpha-diversity using the R package “stats.” Spearman’s correlations between archaeal alpha-diversity and water environmental factors were tested using the R package “psych” (32). Using the R package “vegan,” PCoA based on Bray-Curtis or Sørensen dissimilarity was applied to visualize the compositional variation of archaeal communities across nucleic acid types, months, and zones; permutational multivariate analysis of variance (PERMANOVA) was applied to test the influence of month, zone, nucleic acid type, and their interactions on compositional variation of archaeal communities; and constrained analysis of principal coordinates (CAP) was applied to further visualize the associations of variation in archaeal community composition with environmental factors, with the “vif.cca” function to pre-filter redundant variables (variance inflation factors >10) to avoid the collinearity issues and the “envfit” function to select correlative variables (*P* < 0.05) for generating the final ordinations. Time-decay patterns in Bray-Curtis similarity were fitted to visualize the temporal turnover of archaeal communities within each zone using the R package “stats.” Two-way ANOVA was applied to test the influence of zone, month, and their interaction on water environmental factors, the relative abundance of key archaeal genera in the total community or potentially active assemblages, and the potential activity of key archaeal genera using the R package “stats.” A heatmap was used to visualize Spearman’s correlation coefficients between the relative abundance of key archaeal genera in the total or potentially active communities and water environmental factors, as well as between the potential activity of these genera and their relative abundances or water environmental factors, using the R package “pheatmap” (33). The sequences of the 50 most abundant ZOTUs were aligned using MUSCLE5 (34), trimmed with trimAl (35), and subsequently used to construct a phylogenetic tree with FastTree (36). Heatmaps and bubble plots were used to illustrate the relative dominance and potential activity of these ZOTUs between the lagoon and the seaward zone over the seasonal cycle, as well as their relationships with water environmental factors, using the R packages “pheatmap” and “ggplot2” (33, 37).

## RESULTS AND DISCUSSION

### The artificial lagoon has created a unique water environment

In the context of seasonal variability, this study investigated the impacts of the lagoon project on water environmental conditions and archaeal communities. As noted above, since lagoon operating activities (inlet and discharge) are highly seasonal, seasonality in this study is defined as a compound concept, representing the combined effects of natural climatic variations (such as temperature) and seasonal operating and other human activities. Furthermore, by analyzing the differences in water environmental factors and archaeal communities between the lagoon and the adjacent seaward zone—used as a reference site resembling the Meishan Bay Channel before its transformation into a lagoon (38), as also reflected by the remote sensing images (Fig. S1)—at each sampling time, this study reflects the influence of lagoon project on the water environment and then the archaeal community under seasonal variation. This influence was prominently reflected by the consistently lower salinity in the lagoon waters relative to the seaward waters over the seasonal cycle (Fig. 1b). In summer, the salinity of the lagoon water exhibits stronger fluctuations than that of the seaward water, which may correspond to the high-frequency intervention stage (from June to September) of the lagoon. Water temperature exhibited a subtropical seasonal regime and differed between the two zones for most of the year, being higher in the lagoon than in the seaward zone during spring and summer, but generally lower in two-thirds of the autumn and winter months (Fig. 1b). The seaward waters were generally more turbid than the lagoon waters either by the remote sensing images (Fig. S1) or by SP content in most months (Fig. 1b), which is largely attributable to the sedimentation effect of particulate matters in relatively quiescent and enclosed waters within the lagoon. Furthermore, the limited nutrient input from the open sea may account for the generally lower concentrations of nitrate and phosphate observed in the lagoon waters compared with the seaward zone across most months (Fig. 1b).

The excitation-emission matrix fluorescence spectroscopy with parallel factor analysis identifies three major FDOM components (C1, C2, and C3) corresponding to distinct substances/sources (Fig. S2 and Table S2). Dynamics in the relative quantity of three FDOM components across seasons and zones were observed (Fig. S3a), and the PCA plot showed an overall difference in FDOM composition between the lagoon and the seaward zone, as well as seasonal variations (Fig. S3b). The generally higher humification index—one of the key DOM fluorescence indices—in the lagoon waters compared with the seaward zone across most months (Fig. S4) further confirmed the distinct FDOM composition of the lagoon. Collectively, irrespective of seasonal variability, the artificial lagoon project has created a unique water environment, characterized by more quiescent water masses, reduced salinity, nutrients, and SP, and a distinct FDOM composition compared with the seaward waters. This outcome fulfills, at least in part, one of the original goals of ecological restoration—improving water quality.

### The artificial lagoon overrides seasonal variability in archaeal alpha-diversity

**Fig 2:**
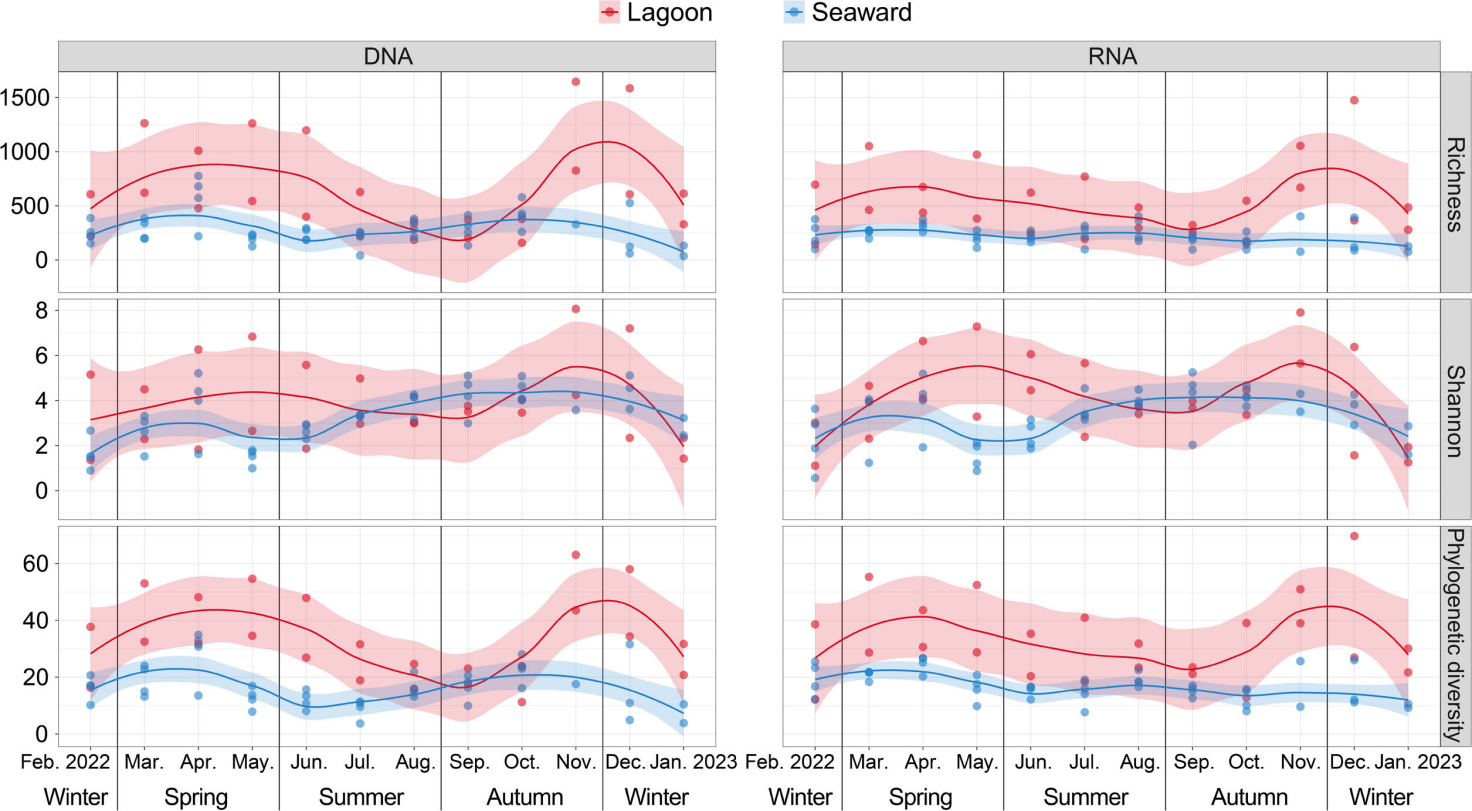
Annual dynamics of alpha-diversity indices of DNA- and RNA-based archaeal communities in the lagoon and seaward waters. Eleven samples with low sequence counts were excluded, and then the ZOTU table was rarefied to 11,542 sequences per sample. The shaded areas represent the 95% confidence intervals for the fitted LOESS curves.

Differences in archaeal alpha-diversity indices—particularly ZOTU richness and phylogenetic diversity—between the lagoon and the seaward zone were generally more pronounced than seasonal variations across months, as reflected by the ANOVA F-values (both *P* ≤ 0.01; Fig. 2 and Table 1). For most months, both alpha-diversity indices were higher in the lagoon than in the seaward waters, despite the lagoon exhibiting more pronounced seasonal fluctuations than the seaward zone (Fig. 2). These patterns were consistent across both DNA- and RNA-based assemblages (Fig. 2), with the influence of nucleic acid type (DNA vs RNA) being minimal and restricted to ZOTU richness (*P* = 0.037; Table 1). In contrast, spatial and seasonal variations were comparable for the Shannon index (Table 1). Our pilot study in this area demonstrated that archaeal relative abundance within the prokaryotic community (using an archaeal-bacterial universal 16S rRNA gene primer set) was much lower in the lagoon than in the seaward waters in the spring (May) of 2019 (21), suggesting that archaeal proliferation may be inhibited within the broader prokaryotic community in the lagoon. Here, the higher archaeal richness and phylogenetic diversity observed in the lagoon across many months (including May) indicate a decoupling between archaeal alpha-diversity and abundance. This pattern further suggests that the majority of archaeal abundance was likely contributed by a few dominant taxa, which is supported by much smaller between-zone differences in the Shannon index, an index reflecting community evenness (39).

**TABLE 1:** Three-way repeated measures ANOVA testing the significance of the influence of zone, month, nucleic acid type (type), and their interactions on alpha-diversity indices of archaeal communities^*a*^

Factor	ZOTU richness		Shannon index		Phylogenetic diversity	
	F	*P*	F	*P*	F	*P*
Zone	64.90	**<0.001**	8.665	**0.004**	106.4	**<0.001**
Month	3.487	**<0.001**	4.848	**<0.001**	4.685	**<0.001**
Type	4.491	**0.037**	0.222	0.639	0.024	0.877
Zone × Month	3.515	**0.001**	2.701	**0.005**	3.115	**0.001**
Zone × Type	0.106	0.745	0.340	0.561	0.012	0.912
Month × Type	0.505	0.895	0.218	0.996	0.396	0.954
Zone × Month × Type	0.475	0.914	0.317	0.980	0.554	0.860

^*a*^ Bold *P* values present significant influence (*P* < 0.05).

For both DNA- and RNA-based assemblages, salinity was negatively correlated, and FDOM C2 positively correlated, with archaeal richness and phylogenetic diversity (*P* ≤ 0.002; Table S3). Meanwhile, SP, temperature, and/or phosphate were positively correlated with the Shannon index of both types of archaeal communities. Accordingly, higher archaeal alpha-diversity was observed in the inshore and nearshore waters, which receive greater anthropogenic and terrestrial inputs, compared with offshore waters at the regional scale (40). Since there is no consensus on how microbial alpha-diversity governs ecosystem functioning, and because microbial diversity-ecosystem function relationships are thought to vary under different anthropogenic pressures (41), alpha-diversity is rather a question than an answer in microbial ecology (42). Therefore, although lagoon maintenance positively influenced archaeal richness and phylogenetic diversity regardless of seasonal variation, its ecological implications for ecosystem functioning remain to be further investigated.

### Unmasked influence of the lagoon on archaeal community composition under strong seasonality

**Fig 3:**
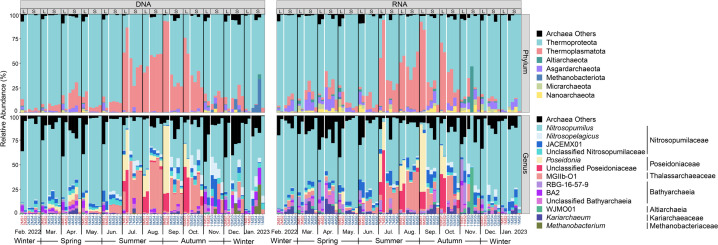
Annual dynamics of taxonomic distribution of DNA- and RNA-based archaeal communities in the lagoon and seaward waters (at the phylum and genus levels, average relative abundance ≥5% at least in one zone in one month). The prefix “unclassified” refers to an archaeal group that has not been classified at the genus level, thus resulting in its identification at higher taxonomic levels. Station IDs colored in red and blue present Meishan Bay Lagoon and Seaward stations, respectively.

In contrast to the patterns observed in archaeal alpha-diversity, the taxonomic distribution of DNA- and RNA-based archaeal communities showed markedly stronger seasonal variation throughout the year than differences between the lagoon and the seaward zone (Fig. 3). Both DNA- and RNA-based archaeal communities were predominated by the phyla Thermoproteota and Thermoplasmatota (Fig. 3). Specifically, Thermoproteota taxa—primarily the Nitrosopumilaceae (MGI) genus *Nitrosopumilus*—overwhelmingly dominated both DNA- (Lagoon: 88.31%, Seaward: 84.53%, in average) and RNA-based (Lagoon: 90.41%, Seaward: 87.38%) communities throughout all winter and spring months. In contrast, Thermoplasmatota taxa—primarily Poseidoniales (MGII)—experienced pronounced blooms during most of the summer and autumn months (July to October), becoming overwhelmingly dominant in the lagoon and substantially abundant in the seaward zone, with average relative abundances of 68.43% and 39.72% in total, and 68.04% and 26.32% in active archaeal communities, respectively. These MGII blooms coincided with the persistent dominance of Thermoproteota, which accounted for 29.21% (Lagoon) and 56.07% (Seaward) of total archaeal communities, and 27.01% (Lagoon) and 64.46% (Seaward) of active communities. During the same periods, niche partitioning between two Poseidoniales families—Poseidoniaceae (MGIIa, mainly the genus *Poseidonia*) and Thalassarchaeaceae (MGIIb, primarily the genus MGIIb-O1)—was apparent between lagoon and seaward waters. Notably, unclassified MGIIa taxa predominated in the lagoon from July to October. These results suggest seasonal niche partitioning—particularly in the lagoon—between the two major planktonic archaeal groups, Nitrosopumilaceae (MGI) and Poseidoniales (MGII). This is consistent with previous studies in the Mediterranean and other coastal sites (18, 20, 43, 44), which reported common MGII blooms in summer. Seasonal niche separation within MGII populations has often been reported; for example, MGIIb (and MGI) and MGIIa typically dominated during winter and summer, respectively (44). However, we did not observe this pattern in either the lagoon or the seaward zone. Instead, our results reveal spatial niche separation within MGII populations between the two zones. Taken together, these findings suggest that niche partitioning between MGI and MGII is likely more season-dependent, whereas niche partitioning within MGII populations appears to be more influenced by sea-use type in the study area. Furthermore, we observed notable differences in the distribution of MGI (also known as AOA) populations between the two zones. The MGI genus *Nitrosopelagicus* became considerably abundant—particularly in the DNA-based community—at most seaward stations during all summer and autumn months, as well as in December and January (Fig. 3). In summary, the pronounced seasonal variation in taxonomic distribution did not mask the influence of the artificial lagoon on archaeal community composition across taxonomic scales.

**Fig 4:**
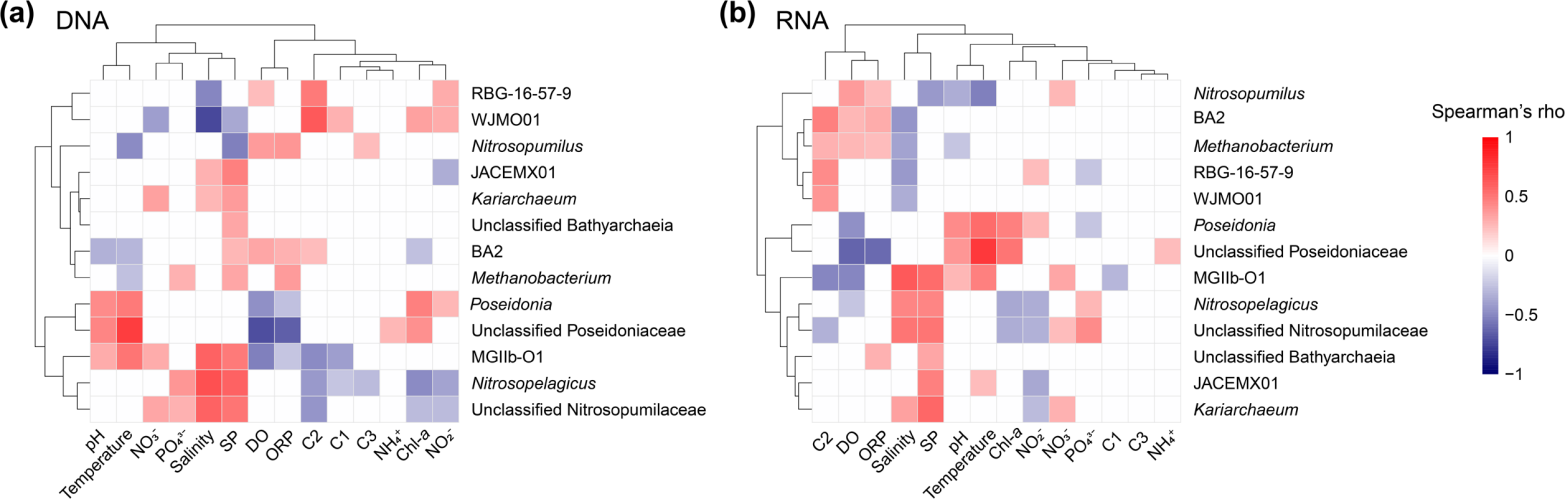
Heatmap illustrating Spearman’s correlation coefficients between the relative abundance of key archaeal genera in the total community (**a**) or potentially active assemblages (**b**) and water environmental factors across the study area. Colored cells indicate significant correlations (*P* < 0.05). The prefix “unclassified” refers to an archaeal group that has not been classified at the genus level, thus resulting in its identification at higher taxonomic levels.

These taxonomic distribution patterns were largely conditioned by the spatiotemporal variability of environmental factors, irrespective of whether the analysis was DNA- or RNA-based (Fig. 4). Specifically, the relative abundance of *Nitrosopumilus* was positively correlated with DO and ORP, while being negatively correlated with SP and temperature. The significant seasonal variations in both the relative abundance of *Nitrosopumilus* and these environmental factors (Table S4 and S5) suggest seasonal fluctuations in environmental conditions likely drove the seasonal dynamics of *Nitrosopumilus*. In contrast, *Nitrosopelagicus* showed positive correlations with salinity, SP, and phosphate, but negative correlations with Chl-*a* and nitrite, in both DNA- and RNA-based communities. As a dominant AOA population in open ocean surface waters globally (45), *Nitrosopelagicus* tends to thrive under salinity levels close to those of oceanic waters (46), which likely explains its frequently observed higher abundance in the seaward waters compared to the lagoon. Furthermore, JACEMX01, recently named as *Candidatus* Nitrosomaritimum (47), a relatively less-known MGI (AOA) genus, exhibited extensive spatial and seasonal prevalence in both DNA- and RNA-based communities, consistent with its widespread distribution in global estuarine-coastal niches (48). In these communities, JACEMX01 showed a strong positive correlation with SP (Fig. 4), which could partially explain its overall higher abundance in the seaward zone compared to the lagoon (Fig. 3). This trend also aligns with its high abundance reported in intertidal aquifers and marine sediments (47), which serve as the primary source of SP in the seaward waters through sediment resuspension in the study area. The relative abundance of two major MGII genera—*Poseidonia* and MGIIb-O1, and unclassified MGIIa taxa—consistently showed positive correlations with temperature and pH, and a negative correlation with DO (Fig. 4). These shared associations suggest that higher temperatures and lower DO levels during the summer and autumn likely triggered the seasonal bloom of MGII, consistent with previous studies (18, 20, 43, 49). Additionally, specific associations of the MGII genera with distinct environmental factors further highlight niche partitioning of MGII populations between the two zones. In both DNA- and RNA-based communities, *Poseidonia* was associated with nitrite and Chl-*a*, and MGIIb-O1 with salinity, SP, nitrate, and FDOM C1 and C2 (Fig. 4). These distinct patterns underscore the ecological specialization of MGII populations across various environmental gradients, even at the local scale. Since MGII taxa are (photo)heterotrophs, as indicated by metagenomic evidence (17, 18), and negative associations were observed between MGIIb-O1 and FDOM components, while *Poseidonia* showed no associations with FDOM components (Fig. 4), FDOM composition likely played a key role in niche partitioning between MGIIa and MGIIb taxa across the lagoon and seaward zones. In addition to the MGI and MGII taxa, Bathyarchaeia—a group known for its extensive taxonomic diversity, carbon metabolic versatility, and prevalence in marine sediments (50)—was occasionally abundant both spatially and seasonally, emerging as the third most abundant archaeal group across the study area, primarily represented by the genera RBG-16-57-9 and BA2, as well as a high proportion of unclassified Bathyarchaeia taxa (Fig. 3). In both DNA- and RNA-based communities, RBG-16-57-9 was mainly associated with FDOM C2 and salinity, BA2 with FDOM C2 and DO, and the unclassified Bathyarchaeia taxa with SP (Fig. 4). The positive associations of RBG-16-57-9 and BA2 with humus-like DOM (C2) reflected their preference for specific carbon sources. Collectively, our findings reveal the key environmental factors driving the seasonal niche differentiation of major archaeal taxa and demonstrate that the artificial lagoon reshapes archaeal communities by modulating environmental conditions.

**Fig 5:**
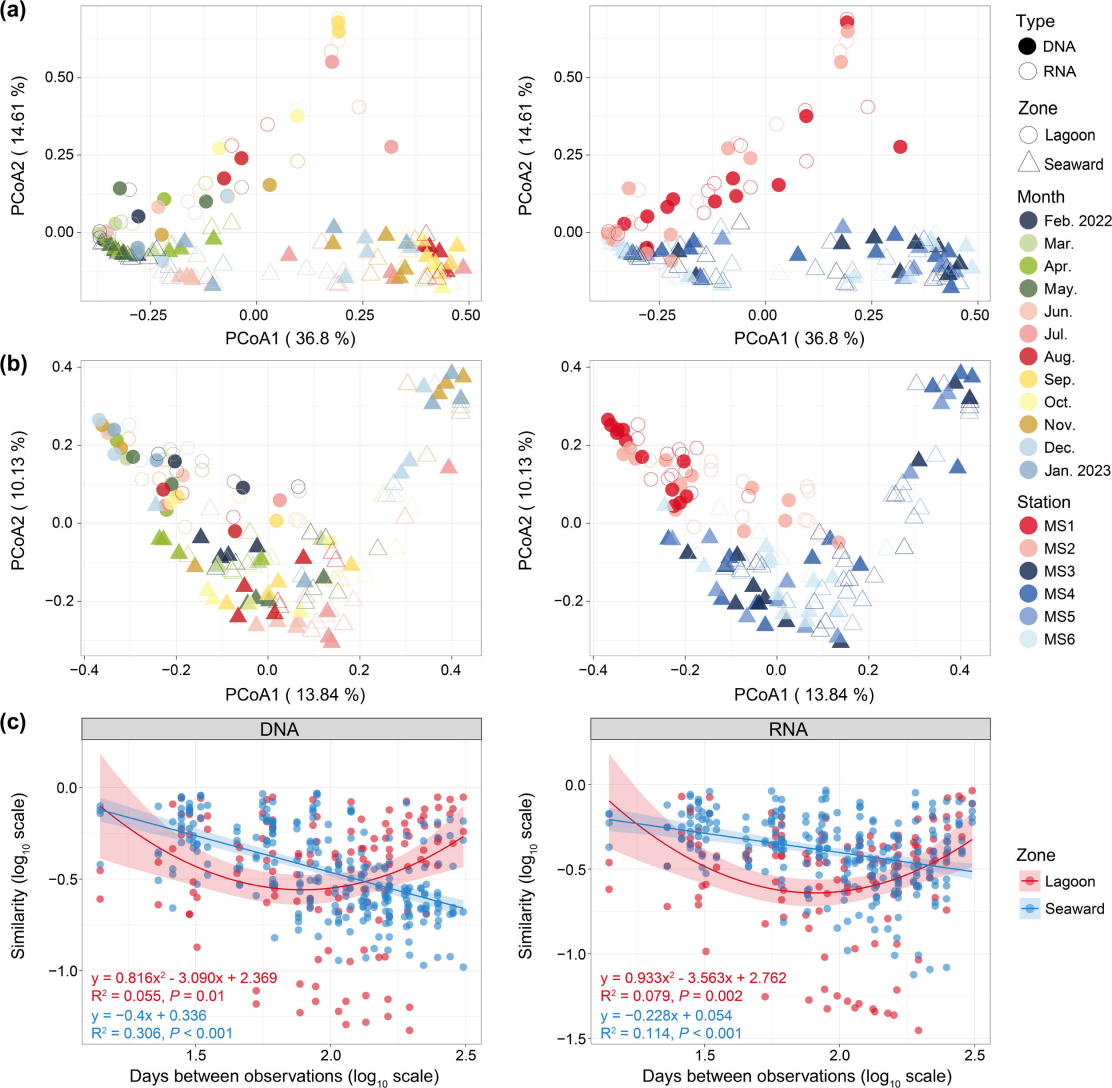
PCoA based on Bray-Curtis (**a**) and Sørensen (**b**) dissimilarity illustrating the compositional variation of archaeal communities across nucleic acid types (type), zones, and months. Time-decay patterns in Bray-Curtis similarity between DNA- or RNA-based archaeal communities in the lagoon and seaward waters (**c**). The data of similarity values and days between observations were shown as log_10_-transformed. Solid and hollow shapes represent DNA- and RNA-based communities, respectively, while different symbols indicate distinct zones, and colors denote months or stations. A linear regression fit was applied to the seaward data (blue), while a quadratic polynomial regression fit was applied to the lagoon data (red) with shaded areas indicating the 95% confidence intervals. The fitted equations, coefficients of determination (adjusted R^2^), and significance levels (*P*) are shown in corresponding colors for each zone.

At a finer taxonomic resolution (based on ZOTUs), archaeal community composition significantly varied across months and zones, irrespective of nucleic acid type or beta-diversity metric (Fig. 5a and b and Table 2). Similar to observations at higher taxonomic ranks, although seasonal variation in archaeal community composition generally exceeded the difference between the lagoon and the seaward zone (Table 2), compositional differences between the two zones were particularly pronounced during specific seasons, namely summer and autumn (Fig. 5a and b). Despite minimal differences in archaeal alpha-diversity between DNA- and RNA-based communities, beta-diversity metrics consistently revealed significant compositional distinctions between total and active communities (all *P*_PERMANOVA_ < 0.01; Table 2). CAP further revealed that seasonal variation in archaeal community composition was largely determined by water temperature, SP, DO, ORP, and/or pH, whereas differences between the two zones were mainly driven by salinity, FDOM C2 (humus-like substance), nitrate, and nitrite, irrespective of nucleic acid type (Fig. S5). In addition to environmental factors such as temperature, SP, DO, salinity, and inorganic nitrogen, which are commonly associated with the spatiotemporal dynamics of archaeal community composition in coastal waters (20, 43, 49), humus-like DOM (C2), often derived from terrestrial organic matter (51), played a key role in creating the unique lagoon conditions that shaped archaeal community composition, particularly during the summer and autumn. Previous work showed that AOA assemblages exhibited much greater stability than ammonia-oxidizing bacteria across various land-use types in coastal wetlands (52), prompting us to explore the temporal turnover of archaeal community composition in response to changes in sea-use types. We observed distinct temporal turnover patterns in archaeal community composition between the lagoon and seaward zones. In the seaward zone, both DNA- and RNA-based archaeal communities consistently exhibited a significant linear time-decay pattern in community similarity (Fig. 5c). In contrast, lagoon archaeal communities showed a nonlinear relationship between community similarity and time, characterized by an initial sharp decline, followed by a subsequent increase. This indicates an accelerated seasonal recurrence, particularly in DNA-based communities, as evidenced by high community similarity between the samples from February 2022 and January 2023 (Fig. 5a and b). This quicker structural recurrence observed in the lagoon is likely attributable to fundamental differences in physical system configuration between the lagoon and the seaward zone. The semi-enclosed nature of the lagoon limits hydrodynamic connectivity despite the routine operating activities, thereby creating a more stable setting for archaeal community reassembly. In contrast, the seaward waters are open and continuously influenced by offshore mixing and advective inputs, exposing microbial communities to greater variability and reducing the potential for structural recurrence. This interpretation is consistent with recent findings showing that lower physical connectivity can enhance microbial structural stability by reducing exposure to external disturbances (53). In conclusion, this study elucidates the influence of the artificial lagoon project on archaeal community composition in the context of seasonal variation, from broader to finer taxonomic levels, as well as in terms of environmental drivers and turnover patterns.

**TABLE 2:** PERMANOVA based on Bray-Curtis and Sørensen dissimilarity (with 999 permutations) quantifying the effects of month, zone, and their interaction on the compositional variation of DNA-based archaeal communities, as well as the effects of month, zone, nucleic acid type (DNA and RNA), and their interactions on the variation in archaeal community composition^*a*^

Data set	Factor	Bray-Curtis		Sørensen	
		R^2^	*P*	R^2^	*P*
DNA	Month	0.436	**0.001**	0.389	**0.001**
	Zone	0.117	**0.001**	0.084	**0.001**
	Month × Zone	0.196	**0.001**	0.206	**0.001**
DNA and RNA	Month	0.392	**0.001**	0.335	**0.001**
	Zone	0.114	**0.001**	0.077	**0.001**
	Type	0.013	**0.001**	0.011	**0.003**
	Month × Zone	0.178	**0.001**	0.174	**0.001**
	Month × Type	0.026	0.697	0.037	0.538
	Zone × Type	0.008	**0.005**	0.008	**0.027**
	Month × Zone × Type	0.019	0.989	0.031	0.927

^*a*^ R^2^ values present the proportion of variance constrained by the factors. Bold *P* values present significant influence (*P* < 0.05).

### Influence of the artificial lagoon on archaeal community assembly processes

In contrast to bacterial communities, the mechanisms underlying archaeal community assembly in marine waters have been relatively understudied. Our previous studies based on null model analyses demonstrated that stochastic processes primarily govern the assembly of archaeal communities in coastal waters at a regional scale in the East China Sea (49, 54). However, the niche partitioning patterns of MGI and MGII taxa appeared to be largely shaped by environmental conditions (49). This decoupling between patterns and processes highlights the complexity and challenges in evaluating the ecological mechanisms that drive community diversity patterns (55). In this study, we applied a framework based on the convergence and divergence of abundance- and incidence-based distances between DNA- and RNA-based assemblages, derived from actual measurements and observations (27), to assess shifts in archaeal community assembly processes across months and zones. This approach does not rely on null model inference, which may introduce bias through over-randomization that deviates from real-world conditions when generating null distributions (56).

**Fig 6:**
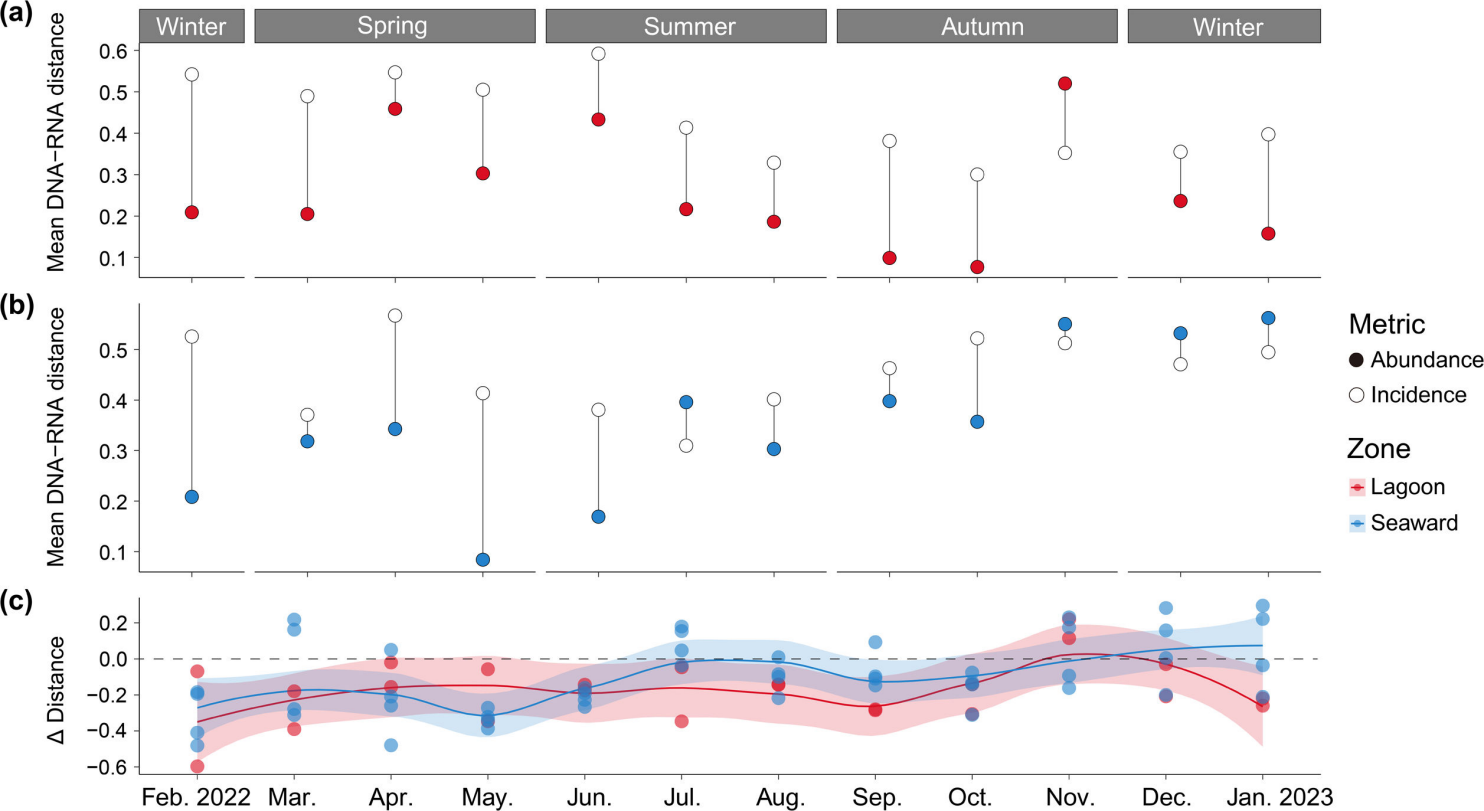
Convergence and divergence patterns of abundance- and incidence-based distances between DNA- and RNA-based assemblages indicating shifts in assembly processes of archaeal communities across seasons and zones. Distances between DNA and RNA of the same sample were averaged by lagoon stations (**a**) and seaward stations (**b**). Fitted LOESS curves of Δ-distances between abundance- and incidence-based distances indicate shifts in the relative importance of selective forces and mass effects in governing archaeal community assembly (**c**). The dashed line at the zero level (y = 0) serves as a reference for identifying the directionality of Δ-distances values and distinguishing between processes driven by selection and by water-mass effects, while the solid lines represent the fitted LOESS curves with shaded areas indicating the 95% confidence intervals.

Although Δ-distances generally showed seasonal shifts across months in both the lagoon and the seaward zone, negative Δ-distance values were observed in the lagoon in most months, except for November (Fig. 6). In contrast, positive Δ-distance values were frequently detected in some seaward stations. This suggests that water-mass effects predominantly governed archaeal community assembly in the lagoon throughout the year, highlighting the importance of dispersal processes via water movements. While dispersal-related processes are often considered stochastic (57, 58), consistent with previous reports (49, 54), passive dispersal driven by water-mass effects can also be deterministic (57), as water movements within the lagoon were partially regulated by operating activities. In particular, the semi-enclosed lagoon system, characterized by limited water exchange with the open sea and longer water retention times, likely amplified the water-mass effect on archaeal community assembly relative to the seaward zone. In contrast, archaeal community assembly in the seaward zone was occasionally more strongly influenced by selective forces than in the lagoon. Collectively, the importance of water-mass effect on community assembly and environmental conditions on community composition suggests that archaeal communities were likely structured by water-mass dynamics and subsequently maintained by the distinct environmental conditions in the two zones.

### Influence of the artificial lagoon on the potential activity of key archaeal taxa

16S rRNA transcript-based amplicon sequencing has been proposed as an important methodology to characterize the active components of a microbiome (24, 26, 59–61). Despite the debated limitations of rRNA as an indicator of microbial metabolic activity in environmental communities, rRNA remains a useful marker for assessing the relative potential activity of microorganisms—reflecting their protein synthesis potential rather than direct metabolic activity—in complex communities (25, 59). While a recent evaluation questioned the validity of this methodology for assessing microbial activity in complex environmental communities—due to the lack of significant compositional differences between RNA- and DNA-based communities (62)—this observation, derived from a limited set of environments or ecosystems, does not conclusively demonstrate the method’s ineffectiveness. In the present study, however, we observed significant compositional differences between RNA- and DNA-based communities, as described above. Considering the methodological limitations and ongoing debate, we followed previous recommendations and interpreted the RNA-based assemblages as representing the potentially active fraction of the community (25).

**Fig 7:**
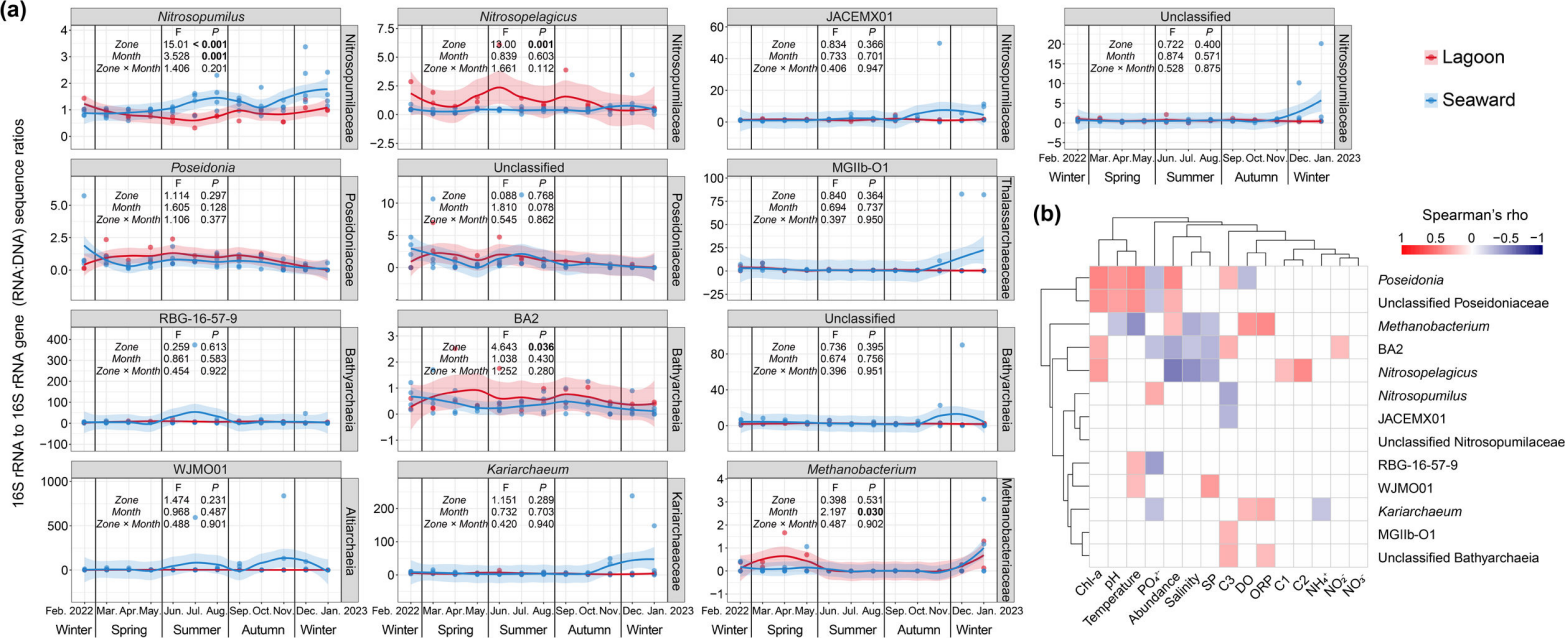
Annual dynamics of potential activity of key archaeal genera (**a**) estimated by 16S rRNA to 16S rRNA gene sequence ratios (RNA:DNA ratios) in the lagoon and seaward waters. The shaded areas represent the 95% confidence intervals for the fitted LOESS curves. Heatmap showing Spearman’s correlation coefficients of RNA:DNA ratios of key archaeal genera with their relative abundances and water environmental factors (**b**). Two-way ANOVA testing the significance of the influence of zone, month, and their interaction on the potential activity of key archaeal genera estimated by RNA:DNA ratios. Bold *P* values indicate significant influence (*P* < 0.05). Colored cells in the heatmap indicate significant correlations (*P* < 0.05). The prefix “unclassified” refers to an archaeal group that has not been classified at the genus level, thus resulting in its identification at higher taxonomic levels. DO, dissolved oxygen; ORP, oxidation-reduction potential; SP, suspended particles; Chl-*a*, chlorophyll *a*; C1–C3, FDOM components 1–3; Abundance, relative abundance.

While the *in situ* activity of archaea in coastal waters remains less extensively characterized than that of bacteria, the narrower range of 16S rRNA gene copy numbers in archaea (1–4) compared to bacteria (1–15) (63) allows for more equitable comparisons of potential activity among archaeal taxa using 16S ratios. The potential activity of the MGI genus *Nitrosopumilus*—the most dominant archaeal group (and AOA) across the study area—varied significantly across zones and months (both *P* < 0.01) (Fig. 7a). It was generally higher in seaward waters than in the lagoon during most summer and winter months, and occasionally during autumn, with zone effects outweighing seasonal variability. In contrast, the potential activity of another MGI genus, *Nitrosopelagicus*, exhibited significant variation only between zones (Fig. 7a). Other major archaeal genera exhibited only subtle differences in potential activity across certain months or between zones within specific months, and no significant overall spatiotemporal trends were observed (Fig. 7a). This aligns with the weaker associations observed between the potential activity of archaeal genera and environmental factors (Fig. 7b), compared to those based on their relative abundances in DNA- and RNA-based communities (Fig. 4). Among MGI genera, the potential activity of *Nitrosopumilus* was positively associated with phosphate and negatively with FDOM C3; *Nitrosopelagicus* was positively associated with FDOM C2 and C1, and Chl-*a*, and negatively with salinity and SP; while JACEMX01 was negatively associated with FDOM C3 (Fig. 7b). The two major AOA genera, *Nitrosopumilus* and *Nitrosopelagicus*, exhibited contrasting responses to lagoon conditions, with *Nitrosopumilus* generally showing lower activity, while *Nitrosopelagicus* occasionally exhibited higher activity. These results underscore the complex ways in which lagoon conditions influence AOA activity, potentially affecting nitrogen cycling, as AOA mediate the critical, rate-limiting step of ammonia oxidation in nitrification (64). Although the potential activity of MGIIa genera was less dynamic than that of MGI genera, the activity of *Poseidonia* and unclassified MGIIa taxa was positively associated with temperature, Chl-*a*, and pH, and/or FDOM C3, and negatively associated with phosphate and/or DO (Fig. 7b). In contrast, the genus MGIIb-O1 showed positive associations only with FDOM C3. The Bathyarchaeia genus BA2 exhibited positive associations with Chl-*a*, FDOM C3, and nitrite, and negative associations with SP, salinity, and phosphate. In contrast, the other two Bathyarchaeia genera showed only sparse associations with environmental factors, consistent with their stable activity levels. Previous studies have suggested that rare taxa, or those with lower abundances, often exhibit higher relative activity within a community, as indicated by 16S ratios (60, 65–67). Consistent with this, groups, such as Asgardarchaeota, Altiarchaeota, Nanoarchaeota, and Micrarchaeota, showed greater spatial and seasonal prevalence and dominance in the RNA-based community compared to the DNA-based community, indicating higher potential activity of these less abundant taxa within the total community (Fig. 3). On the other hand, we observed no consistent relationship between relative abundance and potential activity among major genera (Fig. 7b). Specifically, *Poseidonia*, unclassified MGIIa taxa, and *Methanobacterium* exhibited positive correlations, whereas *Nitrosopelagicus* and the Bathyarchaeia genus BA2 showed negative correlations. Other genera exhibited no clear association.

**Fig 8:**
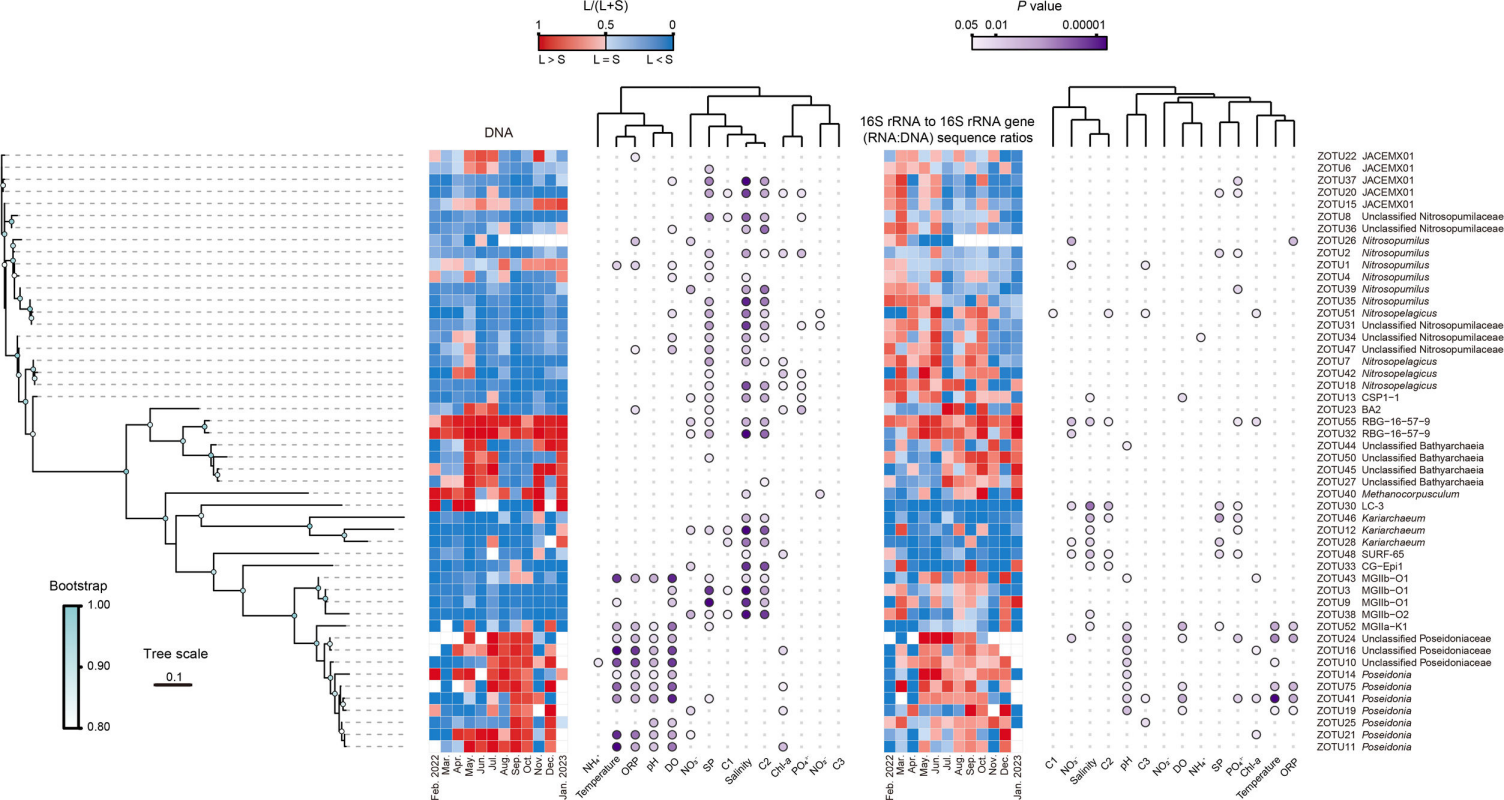
Relative dominance and potential activity of the 50 most abundant archaeal ZOTUs between the lagoon (L) and the seaward zone (S), and their relationships with water environmental conditions. Phylogenetic tree nodes show the bootstrap values greater than 0.8. Heatmaps illustrating relative dominance or activity of these archaeal ZOTUs between the two zones, based on their average relative abundances in the DNA-based community or their average 16S rRNA to 16S rRNA gene sequence ratios. The relative dominance or activity is expressed as the L/(L+S) ratio, where values between 0.5 and 1 (red) indicate greater dominance or activity in the lagoon, a value of 0.5 (black) indicates equality between the two zones, and values between 0 and 0.5 (blue) indicate greater dominance or activity in the seaward zone. Blank cells indicate ZOTUs that were undetected in both zones on a given sampling day, for which the L/(L+S) ratio could not be calculated. Bubble plots show the *P* values of Spearman’s correlations between the relative abundance and potential activity of the archaeal ZOTUs and water environmental factors. Colored bubbles indicate significant correlations (*P* < 0.05). The prefix “unclassified” refers to an archaeal ZOTU that has not been classified at the genus level, thus resulting in its identification at higher taxonomic levels. DO, dissolved oxygen; ORP, oxidation-reduction potential; SP, suspended particles; Chl-*a*, chlorophyll *a*; C1–C3, FDOM components 1–3.

To examine the spatiotemporal variations in relative abundance and potential activity of key archaeal taxa at a finer taxonomic resolution, we selected the top 50 archaeal ZOTUs ranked by relative abundance (accounting for 84.62% of archaeal sequences in the data set) for further analysis (Fig. 8). The distribution of dominant archaeal ZOTUs exhibited a degree of phylogenetic conservatism. AOA ZOTUs belonging to the *Nitrosopumilus* and *Nitrosopelagicus* clusters generally showed higher relative abundance in seaward waters than in the lagoon, except for ZOTU1—the most abundant ZOTU—which exhibited higher relative abundance in the lagoon during roughly half of the months throughout the year. In contrast, other AOA ZOTUs affiliated to JACEMX01 exhibited more frequent alternating dominance between the two zones across months. The ZOTUs within the Bathyarchaeia genus RBG-16-57-9 were generally more abundant in the lagoon than in the seaward zone, whereas other Bathyarchaeia ZOTUs showed reciprocal abundance patterns between the two zones. For MGII ZOTUs, those affiliated to MGIIb were typically more abundant in the seaward zone, while those belonging to the MGIIa cluster alternated in dominance between the two zones over the seasonal cycle. Unlike the distribution patterns of the key archaeal ZOTUs, which are largely shaped by water environmental conditions, the potential activity of ZOTUs across most major archaeal genera exhibited more complex and prevalent alternating dominance between the two zones over the seasonal cycle and was less constrained by environmental factors, consistent with our observations at higher taxonomic ranks. Although seasonal variations in the activity of different ecotypes of MGI, MGIIa, and MGIIb have been reported by others (20), this decoupling between abundance distribution and potential activity was somehow overlooked and can be partially explained by the instantaneous nature of rRNA, which introduces certain stochasticity into RNA-based assemblages. Collectively, our findings suggest that the lagoon project exerts a more pronounced and widespread effect on the distribution of key archaeal taxa than on their potential activity.

### Conclusion

Our work provides a comprehensive assessment of how a coastal lagoon project alters the diversity, community assembly, and potential activity of planktonic archaea. The lagoon project markedly shaped archaeal communities by creating distinct water conditions with reduced salinity and turbidity, along with unique dissolved organic matter profiles. The lagoon’s influence overrode seasonal variability in archaeal alpha-diversity, drove spatial niche partitioning of archaeal taxa—particularly among Poseidoniales populations—despite pronounced seasonal shifts, and promoted higher turnover and more rapid seasonal recurrence in archaeal community composition. While archaeal community assembly is largely governed by water-mass effects, selective forces occasionally play a stronger role in seaward waters. The lagoon project exerted a stronger and more widespread effect on the distribution of key archaeal taxa than on their potential activity, with taxon-specific variations in activity observed among Nitrosopumilaceae genera. Overall, these findings demonstrate that lagoon engineering variably alters archaeal diversity, community assembly, and activity, highlighting the microbial consequences and ecological implications of nearshore restoration projects, and underscoring the importance of integrating microbial perspectives into their planning and evaluation. Since this study was limited to a single year of observations, the interannual variability and reproducibility of archaeal community dynamics, as well as the legacy or cumulative effects of the lagoon project, could not be further evaluated. Long-term, multi-year time-series observations are therefore warranted to address these questions in the future.

## Data Availability

The sequence data have been submitted to the Sequence Read Archive of NCBI under accession number PRJNA1235515 and are accessible with the following link: https://www.ncbi.nlm.nih.gov/sra/PRJNA1235515.

## SUPPLEMENTAL MATERIAL

Supplemental material aem.01499-25-s0001.pdf Supplemental methods, Fig. S1 to S5, and Tables S1 to S5.

ASM does not own the copyrights to Supplemental Material that may be linked to, or accessed through, an article. The authors have granted ASM a non-exclusive, world-wide license to publish the Supplemental Material files. Please contact the corresponding author directly for reuse.
