# Supplementary material for: Artificial lagoon project alters archaeal diversity, community assembly, and potential activity around a nearshore island: insights from an annual cycle
Source: Appl Environ Microbiol. 2025 Dec 30;92(1):e01499-25. doi: 10.1128/aem.01499-25 (PMC12838192; doi:10.1128/aem.01499-25)
Supplement: Supplemental material — Supplemental methods, Fig. S1 to S5, and Tables S1 to S5. [file aem.01499-25-s0001.pdf]

Supplemental Material

Artificial lagoon project alters archaeal diversity, community assembly, and potential activity around a nearshore island: insights from an annual cycle

Haoyu Song[1], Xuya Hu[1], Zhen Chen[2], Lanying Yuan[1], Pengbo Gao[1], Yujie Huang[1], Demin Zhang[1,3], and Kai Wang[1,3*]

[1]Key Laboratory of Aquacultural Biotechnology, Ministry of Education, at School of Marine Sciences, Ningbo University, Ningbo, China

[2]Meishan Bay Tourism Development Service Center in Beilun District, Ningbo, China

[3]Collaborative Innovation Center for Zhejiang Marine High-efficiency and Healthy Aquaculture, Ningbo University, Ningbo, China

[*]For correspondence. E-mail Kai Wang (wangkai@nbu.edu.cn)

Tel. 86-574-87600551; Fax 86-574-87608347.

The supplemental material contains:

- Supplemental Methods
- Supplemental Figures S1-S5
- Supplemental Tables S1-S5

Supplemental Methods

S1. Quantification of fluorescent dissolved organic matters in the waters

The composition of fluorescent dissolved organic matters (FDOM) was measured as previously described (1). Briefly, the water subsamples were firstly filtered through a 0.45-μm glass fiber membrane (Jinjing, China), and then the Excitation-Emission-Matrix (EEM) of FDOM in the filtered samples was measured using a Hitachi F-7000 fluorescence spectrometer (Hitachi, Japan) with scanning ranges of 200-450 nm excitation wavelength and 230-700 nm emission wavelength. Signals were collected in signal-to-reference (S/R) mode with 5-nm and 1-nm intervals for excitation and emission wavelengths, respectively, and a scanning speed of 12,000 nm/min. A Milli-Q water blank was measured along with the samples to eliminate Raman scatter peaks (2).

Parallel Factor Analysis (PARAFAC) of EEMs of water samples was applied to identify FDOM components using MATLAB (MathWorks, USA) with the DOMFluor toolbox as previously described (2). Briefly, we firstly deleted excitation wavelengths from 200 to 245 nm (2). Residual and split half analysis were then performed to validate the identified components. The maximum fluorescence intensity (F_{max}) of a given component was used to represent its relative concentration (3). The emission and excitation wavelengths of the FDOM components were further matched with reference emission and excitation spectra available in the OpenFluor database (https://openfluor.lablicate.com/) (4) to infer the substances/sources of components. The definition and calculation of fluorescence index (FI, an indicator of FDOM source), biological index (BIX, an indicator of FDOM autochthonous origin), and humification index (HIX, an indicator of FDOM humification degree) are provided in Table S1.

S2. Sequence processing

Sequences were processed using USEARCH v11.0.667. Briefly, the paired reads were joined using the script *fastq_mergepairs*. Joined sequences were quality-checked with maximum expected errors (maxEE) = 1.0 using the script *usearch11 -fastq_filter* and then de-replicated using the script *usearch11 -fastx_uniques*. Subsequently, denoise, chimera check and removal, and identification of ZOTUs (Zero-radius Operational Taxonomic Units, 100% sequence similarity cutoff) were processed using UNOISE3 algorithm (5) with the script *usearch11 -unoise3* (minisize = 4). To obtain ZOTU abundances, original joined sequences were mapped to the sequences of ZOTUs at 100% similarity using the script *usearch11 -otutab*. Thereafter, the ZOTU sequences were aligned using Clustal Omega (6), trimmed using trimAl (7), and a phylogenetic tree was then constructed using FastTree (8). The ZOTUs were taxonomically assigned using the Naive Bayes classifier implemented in QIIME 2's q2-feature-classifier plugin (9), based on the SILVA_138.1_SSURef_NR99 reference database (10). To further improve the taxonomic annotation of archaea, the ssu_all_r207 dataset from the Genome Taxonomy Database (GTDB) (11) was also employed. Bacterial, chloroplast, mitochondrial, and unassigned sequences were removed. The full archaeal dataset (including DNA and RNA (cDNA), n = 144) yield 11,265 ZOTUs comprising 6,317,511 qualified reads (read range 604-146,567, mean = 43,871 per sample). To remain as many samples as possible and to ensure fair comparison of ecological communities, different normalizing methods were applied for different analyses. The ZOTU table was normalized via proportions for most downstream analyses as suggested (12) unless otherwise specified. To ensure fair comparisons for alpha-diversity indices, the ZOTU table was rarefied at 11,542 sequences per sample, and 11 samples from November (DNA: D11MS3, D11MS4, and D11MS5; RNA: R11MS3 and R11MS4), December (DNA: D12MS4; RNA: R12MS4), and January (DNA: D1MS4 and D1MS5; RNA: R1MS3 and R1MS5) were excluded, due to their relatively low sequence counts.

Supplemental Figures

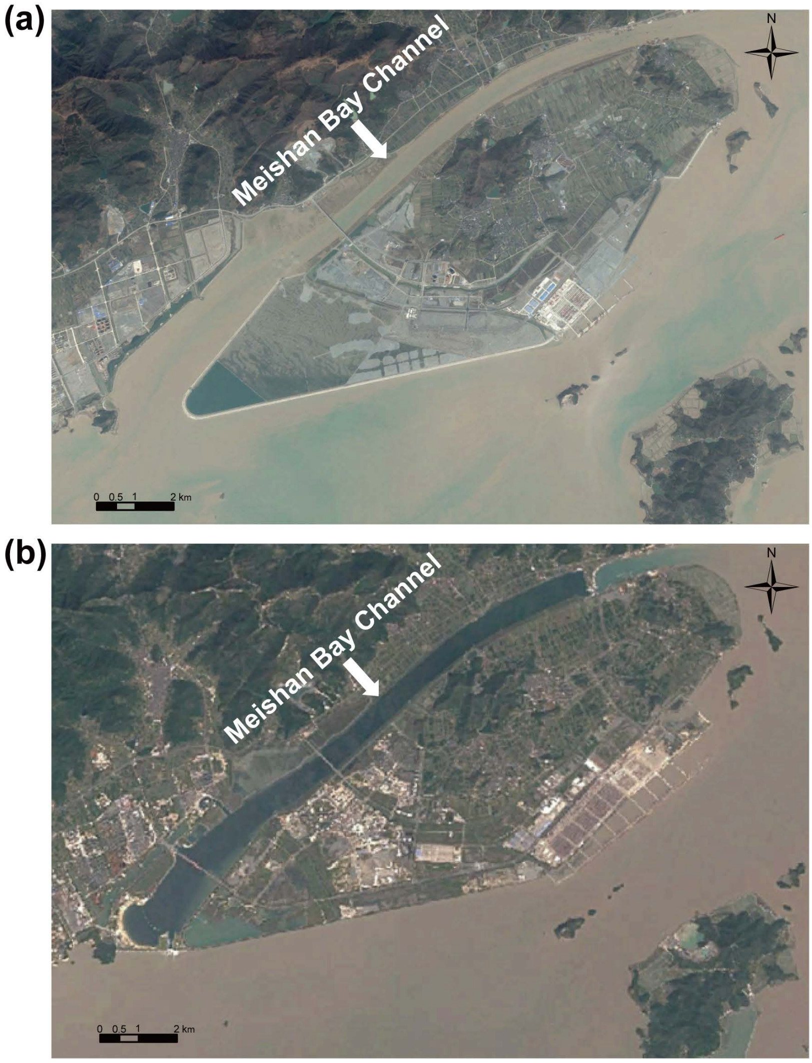

Figure S1. Remote sensing images illustrating the apparent changes in the water environment of the Meishan Bay Channel before (a, December 2012) and 6 years after the construction of the artificial lagoon (b, December 2022, the sampling year). Map data: Google, CNES/Airbus, Maxar Technologies, and Landsat/Copernicus.

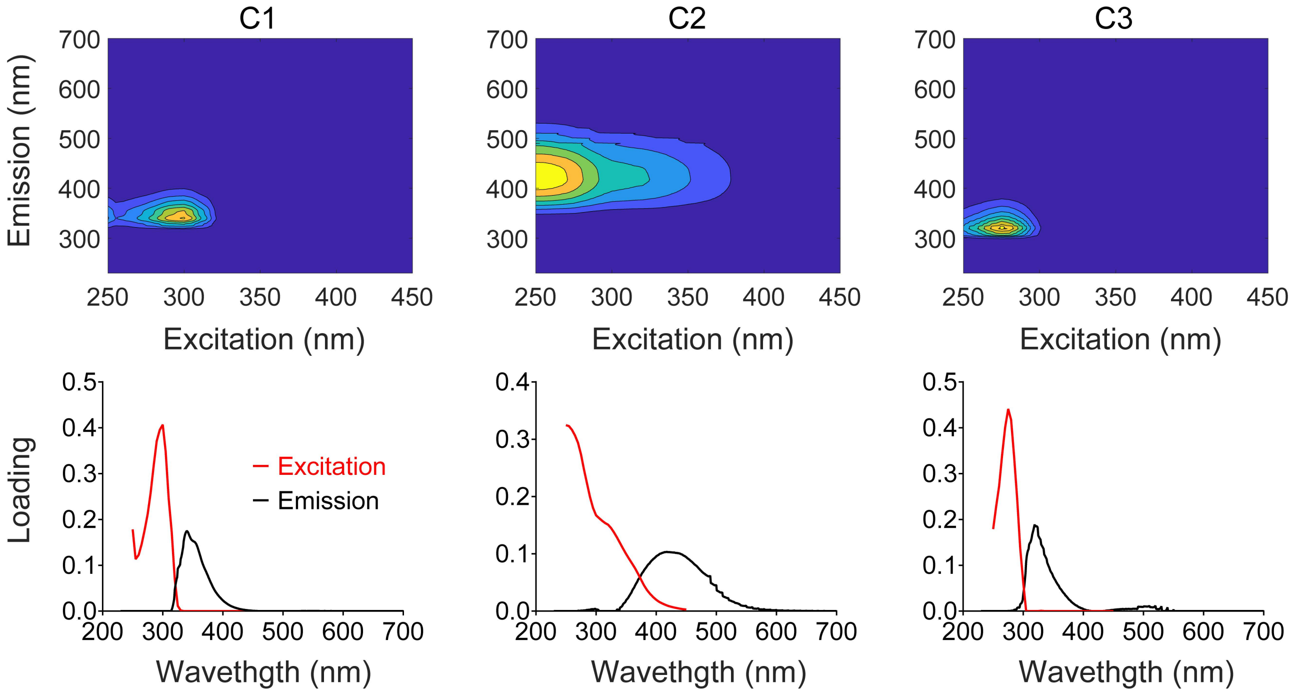

Figure S2. Three-dimensional fluorescence spectra (upper) and maximum excitation/emission wavelength distribution (bottom) of the three FDOM components (C1, C2, and C3; see details in Table S2).

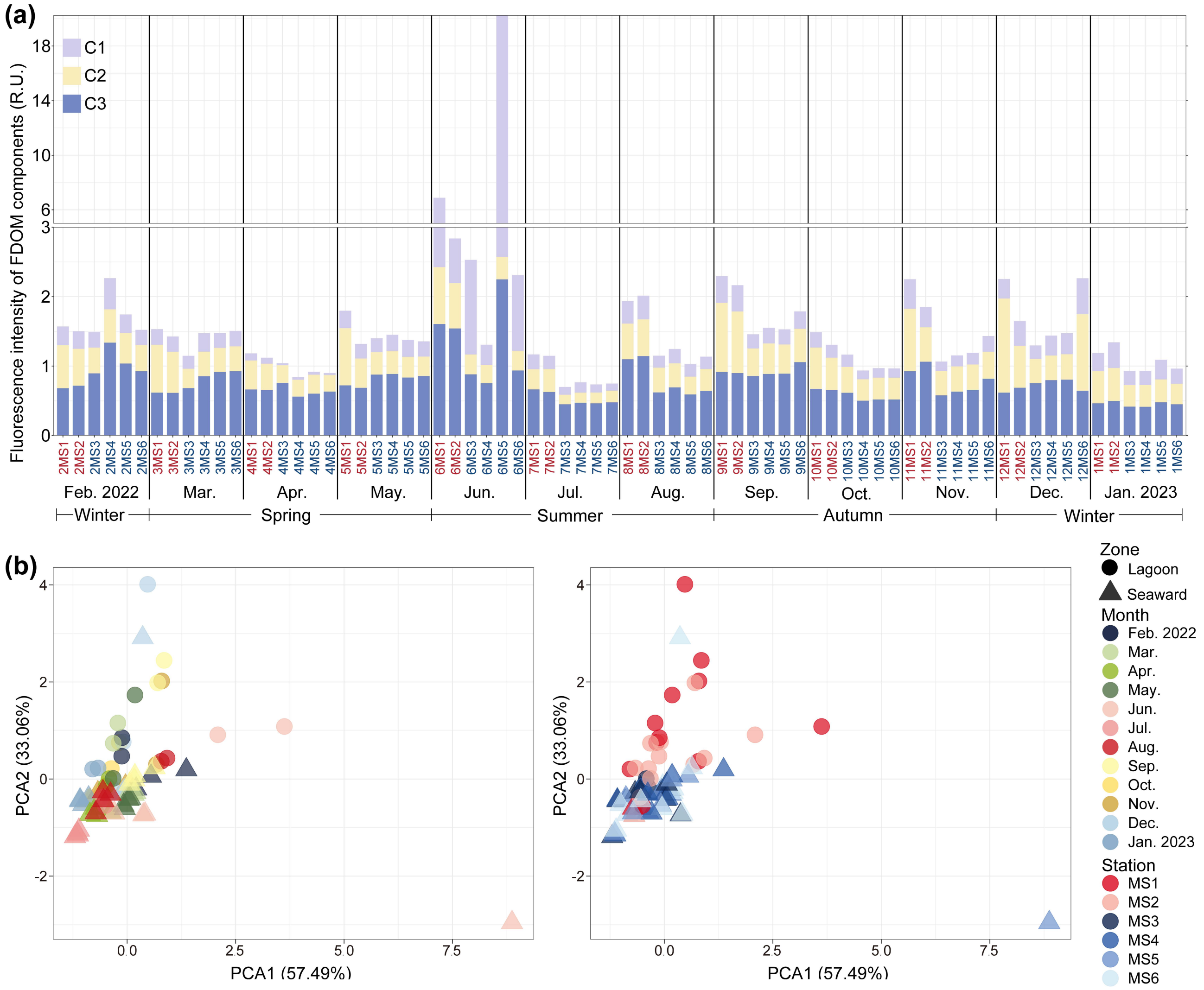

Figure S3. Annual dynamics of FDOM compositions in the lagoon and seaward waters. Spatiotemporal variations in the fluorescence intensity of the three FDOM components (a). Station IDs colored by red and blue represent the lagoon and seaward stations, respectively. Principle Component Analysis (PCA) illustrating the compositional variation of FDOM across months or zones (b).

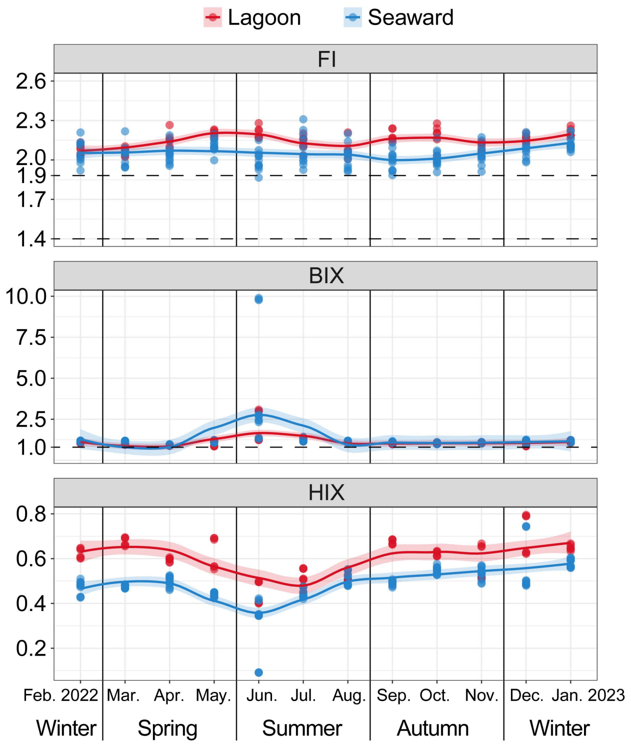

Figure S4. Annual dynamics of FDOM fluorescence indices in the lagoon and seaward waters. FI, fluorescence index; BIX, biological index; HIX, humification index. The dashed lines serve as reference values (see details in Table S1). The shaded areas represent the 95% confidence intervals for the fitted LOESS curve.

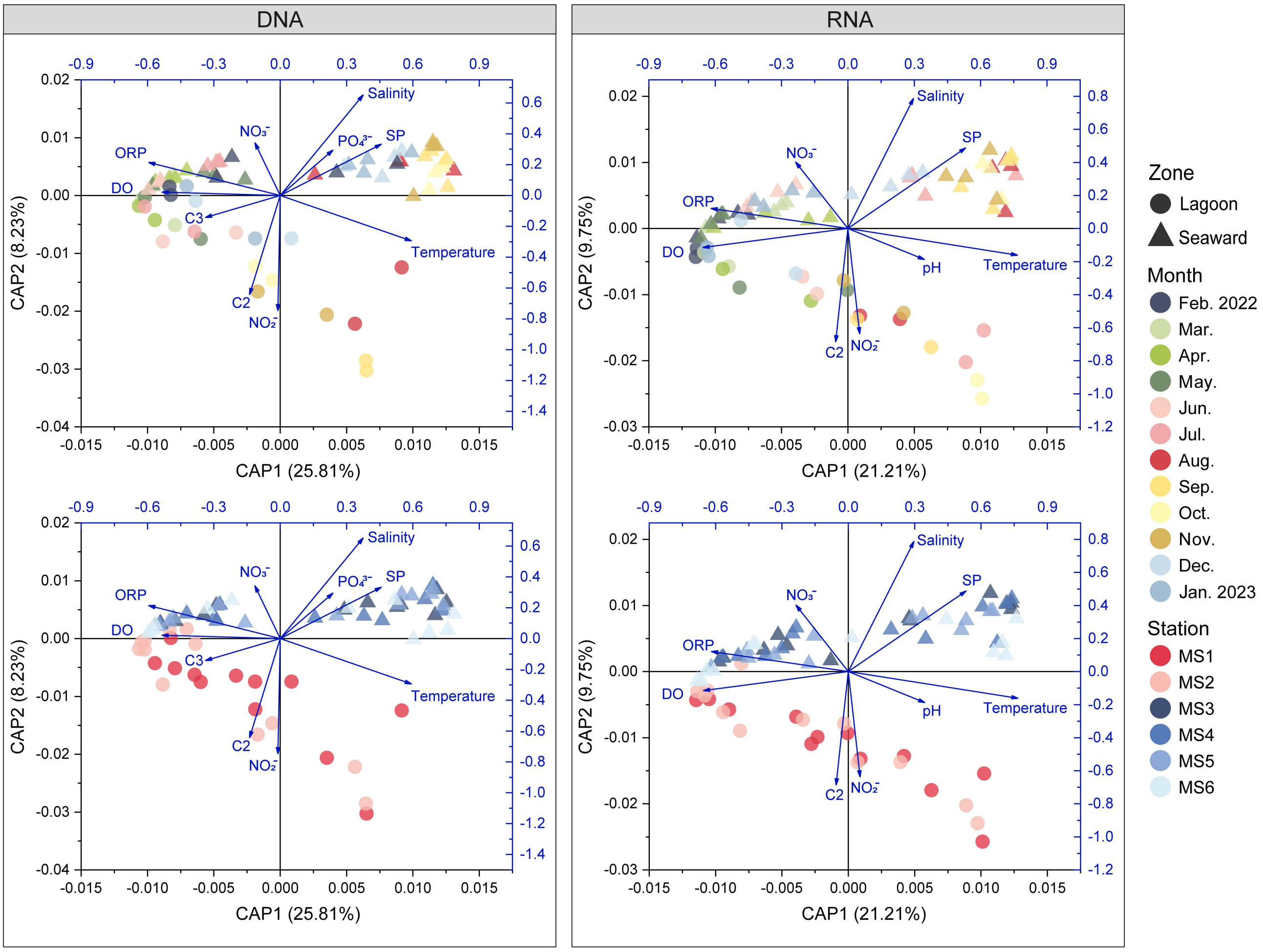

Figure S5. Constrained Analysis of Principal Coordinates (CAP) of DNA- and RNA-based archaeal communities. Environmental variables were co-plotted to identify key environmental factors that drove the compositional variation of archaeal communities. Environmental variables were normalized to reduce the effect of unit differences. DO, dissolved oxygen; SP, suspended particles; C2, FDOM fluorescent component 2; C3, FDOM fluorescent component 3; ORP, oxidation-reduction potential.

Supplemental Tables

Table S1. The definition and calculation of FDOM-associated indies.

Index	Definition	Calculation	Reference
Fluorescence index (FI)	FI has been used to distinguish DOM derived from microbial and terrestrial sources. High values (>1.9) are characteristic of microbially derived fulvic acids (autochthonous origin), whereas low values (<1.4) indicate terrestrially derived fulvic acids (allochthonous origin). Other FI values (1.4-1.9) indicate that DOM originates from a mixture of terrestrial and autochthonous sources.	FI was calculated as the ratio of emission intensity at 450 nm to that at 500 nm, obtained at an excitation wavelength of 370 nm. $FI = \frac{I_{450}}{I_{370}}$	(13)
Biological index (BIX)	BIX has been used to estimate autochthonous biological activity in estuarine and marine samples. High values (>1.0) indicate freshly produced DOM from microbial sources; values (0.8-1.0) suggest strong internal input with minor terrestrial influence; values (0.7-0.8) represent mixed sources; and values (0.6-0.7) indicate a predominance of terrestrial or external input.	BIX was calculated as the ratio of emission intensity at 380 nm to that at 430 nm, obtained at an excitation wavelength of 310 nm. $BIX = \frac{I_{380}}{I_{430}}$	(14, 15)
Humification index (HIX)	HIX has been used to quantify the complexity and aromaticity of DOM whose molecular structures undergo gradual alteration during microbial processes (i.e., degree of humification). High values (>16) suggest strong humic character with substantial terrestrial input; values (6-10) indicate pronounced humic character with weak recent autochthonous contribution; values (4-6) reflect weak humic character alongside a notable recent autochthonous component; and values (<4) correspond to freshly produced autochthonous DOM of aquatic biological or microbial origin.	HIX was calculated as the area under the emission spectra 435-480 nm divided by the peak area under the emission spectra 300-345 and 435-480 nm, at excitation wavelength 254 nm. $HIX = \frac{\sum I_{435\to480}}{\sum I_{300\to345} + \sum I_{435\to480}}$	(14, 16)

Table S2. Characteristics of the EEM-PARAFAC components.

Component	λ_{Ex}/nm	λ_{Em}/nm	Substance/Source
C1	< 225，(300)	342	protein-like (17)
C2	250	430	humus-like substance (18)
C3	275	325	protein-like, tryptophan like (19)

Table S3. Spearman's correlation coefficients between alpha-diversity indices of DNA- and RNA-based archaeal communities and water environmental factors across the entire study area.

Factor	DNA						RNA					
	ZOTU Richness		Shannon index		Phylogenetic diversity		ZOTU Richness		Shannon index		Phylogenetic diversity	
	ρ	*P*	ρ	*P*	ρ	*P*	ρ	*P*	ρ	*P*	ρ	*P*
Salinity	-0.380	**0.002**	-0.048	0.705	-0.520	**< 0.001**	-0.524	**< 0.001**	-0.055	0.660	-0.591	**< 0.001**
C2	0.377	**0.002**	0.161	0.197	0.550	**< 0.001**	0.401	**0.001**	0.173	0.161	0.489	**< 0.001**
NO_3^-	-0.178	0.154	-0.155	0.215	-0.257	**0.037**	-0.190	0.124	-0.242	**0.049**	-0.199	0.107
ORP	0.173	0.165	-0.168	0.177	0.195	0.116	0.172	0.164	-0.094	0.448	0.237	0.053
DO	0.137	0.272	-0.163	0.192	0.188	0.132	0.132	0.288	-0.195	0.114	0.184	0.137
C3	-0.112	0.373	-0.188	0.131	0.001	0.991	0.019	0.877	-0.038	0.762	0.119	0.338
NH_4^+	0.110	0.379	0.116	0.355	0.030	0.810	0.079	0.525	0.116	0.351	0.057	0.648
NO_2^-	0.103	0.409	0.023	0.854	0.137	0.273	0.278	**0.023**	-0.029	0.818	0.283	**0.020**
Temperature	-0.064	0.608	0.258	**0.036**	-0.136	0.275	-0.024	0.844	0.324	**0.007**	-0.098	0.429
SP	-0.063	0.613	0.461	**< 0.001**	-0.168	0.176	-0.233	0.057	0.464	**< 0.001**	-0.230	0.061
PO_4^{3-}	0.038	0.759	0.298	**0.015**	-0.016	0.898	-0.019	0.879	0.169	0.172	-0.039	0.752
Chl-*a*	0.007	0.957	-0.039	0.753	0.010	0.937	0.216	0.079	-0.023	0.853	0.173	0.162
pH	-0.003	0.984	0.099	0.430	-0.034	0.789	-0.027	0.825	0.110	0.377	-0.061	0.623
C1	0.000	0.999	-0.156	0.212	0.104	0.405	0.206	0.094	-0.176	0.154	0.236	0.054

Bold *P* values present significance ($P < 0.05$). DO, dissolved oxygen; SP, suspended particles; ORP, oxidation-reduction potential; Chl-*a*, chlorophyll *a*; C1, FDOM component 1 (protein-like); C2, FDOM component 2 (humus-like substance); C3, FDOM component 3 (protein-like).

Table S4. Two-way Analysis of Variance (ANOVA) testing the significance of the influence of zone, month, and their interaction on water environmental factors.

Factor	*Zone*		*Month*		*Zone × Month*	
	F	*P*	F	*P*	F	*P*
Temperature	15.80	< **0.001**	455.2	< **0.001**	14.10	< **0.001**
Salinity	215.5	< **0.001**	3.939	< **0.001**	3.219	**0.002**
pH	0.078	0.782	4.360	< **0.001**	0.825	0.616
DO	11.88	**0.001**	16.58	< **0.001**	4.702	< **0.001**
SP	9.203	**0.004**	1.625	0.122	0.616	0.806
ORP	2.640	0.111	19.84	< **0.001**	0.709	0.724
NH_4^+	3.173	0.081	6.217	< **0.001**	1.794	0.081
NO_2^-	115.5	< **0.001**	14.88	< **0.001**	28.34	< **0.001**
NO_3^-	35.23	< **0.001**	6.042	< **0.001**	2.415	**0.018**
PO_4^{3-}	1.822	0.183	1.589	0.133	1.582	0.135
Chl-*a*	2.714	0.106	2.180	**0.032**	0.321	0.977
C1	0.079	0.780	1.791	0.082	0.169	0.999
C2	64.97	< **0.001**	4.920	< **0.001**	1.142	0.351
C3	1.448	0.235	8.585	< **0.001**	2.290	**0.024**

Bold *P* values present significant influence ($P < 0.05$). DO, dissolved oxygen; SP, suspended particles; ORP, oxidation-reduction potential; Chl-*a*, chlorophyll *a*; C1, FDOM component 1 (protein-like); C2, FDOM component 2 (humus-like substance); C3, FDOM component 3 (protein-like).

Table S5. Two-way Analysis of Variance (ANOVA) testing the significance of the influence of zone, month, and their interaction on the relative abundance of key archaeal genera in the total community or potentially active assemblages.

Dataset	Taxa	*Zone*		*Month*		*Zone × Month*	
		F	*P*	F	*P*	F	*P*
DNA	*Nitrosopumilus*	0.944	0.336	8.703	**< 0.001**	2.887	**0.005**
	Nitrosopelagicus	86.17	**< 0.001**	11.96	**< 0.001**	5.828	**< 0.001**
	JACEMX01	1.287	0.262	1.834	0.074	0.933	0.518
	Unclassified Nitrosopumilaceae	47.63	**< 0.001**	1.370	0.218	0.952	0.501
	Poseidonia	250.2	**< 0.001**	41.84	**< 0.001**	77.35	**< 0.001**
	Unclassified Poseidoniales	78.22	**< 0.001**	11.55	**< 0.001**	17.06	**< 0.001**
	MGIIb-O1	73.01	**< 0.001**	21.58	**< 0.001**	7.936	**< 0.001**
	RBG-16-57-9	33.38	**< 0.001**	1.701	0.102	2.338	**0.021**
	BA2	0.295	0.589	2.000	**0.049**	0.882	0.563
	Unclassified Bathyarchaeia	0.000	0.998	0.927	0.523	0.762	0.675
	WJMO01	66.59	**< 0.001**	3.268	**0.002**	6.538	**< 0.001**
	Kariarchaeum	6.920	**0.011**	2.028	**0.046**	1.031	0.435
	Methanobacterium	1.573	0.216	1.403	0.202	0.709	0.724
RNA	*Nitrosopumilus*	15.79	**< 0.001**	6.988	**< 0.001**	2.605	**0.011**
	Nitrosopelagicus	22.20	**< 0.001**	4.114	**< 0.001**	2.038	**0.045**
	JACEMX01	1.794	0.187	1.126	0.363	1.947	0.056
	Unclassified Nitrosopumilaceae	34.36	**< 0.001**	1.212	0.305	0.749	0.687
	Poseidonia	383.3	**< 0.001**	49.85	**< 0.001**	92.06	**< 0.001**
	Unclassified Poseidoniales	47.26	**< 0.001**	6.029	**< 0.001**	9.670	**< 0.001**
	MGIIb-O1	43.78	**< 0.001**	10.71	**< 0.001**	4.537	**< 0.001**
	RBG-16-57-9	113.8	**< 0.001**	6.480	**< 0.001**	12.69	**< 0.001**
	BA2	23.19	**< 0.001**	1.427	0.192	2.609	**0.011**
	Unclassified Bathyarchaeia	0.422	0.519	2.276	**0.025**	2.697	**0.009**
	WJMO01	0.042	0.838	1.325	0.241	0.447	0.926
	Kariarchaeum	11.04	**0.002**	1.232	0.292	1.027	0.438
	Methanobacterium	8.278	**0.006**	1.003	0.458	2.119	**0.037**

Bold *P* values present significant influence ($P < 0.05$). The prefix "unclassified" refers to an archaeal group that has not been classified at the genus level, thus resulting in its identification at higher taxonomic levels.

Supplemental Reference

1. Niloy NM, Habib SA, Islam MI, Haque MM, Shammi M, Tareq SM. 2023. Distribution, characteristics and fate of fluorescent dissolved organic matter (FDOM) in the Bay of Bengal. *Mar Pollut Bull* 195:115467. https://doi.org/10.1016/j.marpolbul.2023.115467
2. Stedmon CA, Bro R. 2008. Characterizing dissolved organic matter fluorescence with parallel factor analysis: a tutorial. *Limnol Oceanogr Methods* 6:572-579. https://doi.org/10.4319/lom.2008.6.572
3. Phong DD, Hur J. 2015. Insight into photocatalytic degradation of dissolved organic matter in UVA/TiO2 systems revealed by fluorescence EEM-PARAFAC. *Water Res* 87:119-126. https://doi.org/10.1016/j.watres.2015.09.019
4. Murphy KR, Stedmon CA, Wenig P, Bro R. 2014. OpenFluor- an online spectral library of auto-fluorescence by organic compounds in the environment. *Anal Methods* 6:658-661. https://doi.org/10.1039/C3AY41935E
5. Edgar RC. 2016. UNOISE2: improved error-correction for Illumina 16S and ITS amplicon sequencing. *bioRxiv*. https://doi.org/10.1101/081257
6. Sievers F, Higgins DG. 2014. Clustal Omega, accurate alignment of very large numbers of sequences. *Methods Mol Biol* 1079:105-116. https://doi.org/10.1007/978-1-62703-646-7_6
7. Capella-Gutiérrez S, Silla-Martínez JM, Gabaldón T. 2009. TrimAl: a tool for automated alignment trimming in large-scale phylogenetic analyses. *Bioinformatics* 25:1972-1973. https://doi.org/10.1093/bioinformatics/btp348
8. Price MN, Dehal PS, Arkin AP. 2010. FastTree 2 – approximately maximum-likelihood trees for large alignments. *PLoS One* 5:e9490. https://doi.org/10.1371/journal.pone.0009490
9. Bokulich NA, Kaehler BD, Rideout JR, Dillon M, Bolyen E, Knight R, Huttley GA, Gregory Caporaso J. 2018. Optimizing taxonomic classification of marker-gene amplicon sequences with QIIME 2's q2-feature-classifier plugin. *Microbiome* 6:90. https://doi.org/10.1186/s40168-018-0470-z
10. Quast C, Pruesse E, Yilmaz P, Gerken J, Schweer T, Yarza P, Peplies J, Glöckner FO. 2013. The SILVA ribosomal RNA gene database project: improved data processing and web-based tools. *Nucleic Acids Res* 41:D590-D596. https://doi.org/10.1093/nar/gks1219
11. Parks DH, Chuvochina M, Rinke C, Mussig AJ, Chaumeil PA, Hugenholtz P. 2022. GTDB: an ongoing census of bacterial and archaeal diversity through a phylogenetically consistent, rank normalized and complete genome-based taxonomy. *Nucleic Acids Res* 50:D785-D794. https://doi.org/10.1093/nar/gkab776
12. McKnight DT, Huerlimann R, Bower DS, Schwarzkopf L, Alford RA, Zenger KR. 2018. Methods for normalizing microbiome data: an ecological perspective. *Methods Ecol Evol* 10:389-400. https://doi.org/10.1111/2041-210x.13115
13. McKnight DM, Boyer EW, Westerhoff PK, Doran PT, Kulbe T, Andersen DT. 2001. Spectrofluorometric characterization of dissolved organic matter for indication of precursor organic material and aromaticity. *Limnol Oceanogr* 46:38-48. https://doi.org/10.4319/lo.2001.46.1.0038
14. Huguet A, Vacher L, Relexans S, Saubusse S, Froidefond JM, Parlanti E. 2009. Properties of fluorescent dissolved organic matter in the Gironde Estuary. *Org Geochem* 40:706-719. https://doi.org/10.1016/j.orggeochem.2009.03.002
15. Parlanti E, Wörz K, Geoffroy L, Lamotte M. 2000. Dissolved organic matter fluorescence spectroscopy as a tool to estimate biological activity in a coastal zone submitted to anthropogenic inputs. *Org Geochem* 31:1765-1781. https://doi.org/10.1016/S0146-6380(00)00124-8
16. Ohno T. 2002. Fluorescence inner-filtering correction for determining the humification index of dissolved organic matter. *Environ Sci Technol* 36:742-746. https://doi.org/10.1021/es0155276

17. Calderó-Pascual M, Yıldız D, Yalçın G, Metin M, Yetim S, Fiorentin C, Andersen MR, Jennings E, Jeppesen E, Ger KA, Beklioğlu M, McCarthy V. 2022. The importance of allochthonous organic matter quality when investigating pulse disturbance events in freshwater lakes: a mesocosm experiment. *Hydrobiologia* 849:3905-3929. https://doi.org/10.1007/s10750-021-04757-w
18. Panettieri M, Guigue J, Chemidlin Prevost-Bouré N, Thévenot M, Lévêque J, Le Guillou C, Maron PA, Santoni AL, Ranjard L, Mounier S, Menasseri S, Viaud V, Mathieu O. 2020. Grassland-cropland rotation cycles in crop-livestock farming systems regulate priming effect potential in soils through modulation of microbial communities, composition of soil organic matter and abiotic soil properties. *Agric Ecosyst Environ* 299:106973. https://doi.org/10.1016/j.agee.2020.106973
19. Gao Z, Guéguen C. 2018. Distribution of thiol, humic substances and colored dissolved organic matter during the 2015 Canadian Arctic GEOTRACES cruises. *Mar Chem* 203:1-9. https://doi.org/10.1016/j.marchem.2018.04.001
